# RINAMI: Residue‐attributed interpretable neural network for predicting absolute folding free energy by merging structure and sequence information

**DOI:** 10.1002/pro.70717

**Published:** 2026-07-10

**Authors:** Naoki Tomita, George Chikenji

**Affiliations:** ^1^ Department of Applied Physics, Graduate School of Engineering Nagoya University Nagoya Aichi Japan

**Keywords:** protein stability, machine learning, protein language model

## Abstract

Recent advances in de novo protein design have enabled the generation of diverse novel proteins. However, a fundamental challenge remains: even when an amino acid sequence is designed with the target structure as the most stable conformation, there is currently no reliable computational method for assessing whether the target structure is sufficiently stabilized relative to alternative conformations. While experimental realization of the intended fold requires the target structure to be thermodynamically favored by a large free‐energy gap, the absence of a quantitative measure of folding stability makes it difficult to distinguish reliable from unreliable designs. Here, we propose the Residue‐attributed Interpretable Neural network for predicting Absolute folding free energy by Merging structure and sequence Information (RINAMI), a machine learning model that predicts the absolute folding free energy (Δ*G*) of proteins from their three‐dimensional structures and amino acid sequences. RINAMI integrates structure‐ and sequence‐based representations derived from ProteinMPNN and Evolutionary Scale Modeling 2 (ESM2) using a multi‐head cross‐attention mechanism that contextualizes sequence‐derived signals within the structural environment. Benchmarking RINAMI on both natural and designed proteins from the Mega‐scale and Maxwell datasets shows that it outperforms the tested existing approaches, achieving higher correlations with experimental measurements and improved or comparable prediction errors. An ablation study supports the contribution of sequence–structure integration for predictive accuracy. In addition, RINAMI exhibits strong interpretability by capturing key physicochemical effects, including the destabilizing effect of buried hydrophilic residues, the stabilizing effect of buried hydrophobic residues, and the characteristics of cysteine. Together, these results establish RINAMI as an accurate and interpretable framework for Δ*G* prediction and provide a practical computational tool for evaluating and prioritizing protein designs prior to experimental testing.

## 1 INTRODUCTION

Recent advances in machine learning (ML)‐based tools for de novo protein design and elucidation of the design rules have enabled the creation of numerous novel proteins (Chu et al., 2024; Dauparas et al., 2022; Koga et al., 2012; Lin et al., 2015; Marcos et al., 2018; Minami et al., 2023; Murata et al., 2024; Watson et al., 2023). Yet, despite these technological breakthroughs, experimental success rates in de novo protein design remain low—often below 10% (Lauko et al., 2025; Liu et al., 2025; Torres et al., 2025). A major reason for the low success rate is that many designed sequences fail to stably adopt the intended structure, leading to unstable expression, aggregation, and related experimental failures. Therefore, improving the efficiency and reliability of protein design requires predicting, before experimentation, whether the target structure is thermodynamically favored by a sufficiently large free‐energy gap between the folded state and alternative conformations. This stability is commonly quantified by the free energy difference.

$$\Delta G\equiv G_{\text{unfolded}}-G_{\text{target}}\;\left(\text{kcal}/\mathrm{mol}\right).$$

Thus, computationally predicting the Δ*G* of the target structure of a designed protein is an important objective in protein design.

Despite its central importance, Δ*G* prediction remains intrinsically challenging, as it reflects a complex sum of atomic interactions among backbone and side chains, which depend on protein size, three‐dimensional geometry, sequence context, and solution conditions. Consequently, no generally reliable and broadly applicable indicator or tool for Δ*G* prediction has yet been established (Garcia et al., 2025). This limitation is evident even in state‐of‐the‐art structural prediction tools such as AlphaFold2 (AF2) (Jumper et al., 2021), which provide little to no information about protein stability. Indeed, confidence scores for predicted structures, including predicted local distance difference test (pLDDT) and predicted TM‐score (pTM), have been reported to show no correlation with experimentally measured Δ*G* (Kim et al., 2022). In addition, our previous study reported that a substantial number of predicted structures with high pLDDT values exhibited large conformational fluctuations in molecular dynamics (MD) simulations, indicating that high pLDDT does not necessarily imply thermodynamic stability (Tomita et al., 2025). Although several ML‐based approaches have recently been explored for Δ*G* prediction, further improvements in accuracy and robustness are still needed for practical use. Among these approaches, the most recent and promising method is that proposed by Cagiada et al., which is based on ESM‐IF (Hsu et al., 2022), where IF denotes inverse folding. ESM‐IF is an ML model designed to predict amino acid sequences compatible with a given backbone structure. While this method enables approximate estimation of Δ*G* for various natural proteins (Cagiada et al., 2025), it shows limited accuracy for variant proteins and limited sensitivity to stability changes induced by amino acid mutations, as shown in Figure S1. Taken together, the establishment of a more robust Δ*G* prediction method, applicable to various proteins and their variants, has not yet been achieved and remains necessary.

These observations highlight the need to incorporate sensitivity to mutation‐induced Δ*G* changes into existing Δ*G* prediction methods. To address this need, our study focuses on leveraging amino acid sequence representations derived from protein language models (PLMs), which encode amino acid sequences into vector representations that capture the physicochemical properties of individual residues (Rives et al., 2021). This approach is motivated by previous fine‐tuning studies demonstrating that PLMs achieve high accuracy in predicting mutation‐induced changes in Δ*G* from amino acid sequences (Barducci et al., 2025; Savojardo et al., 2025). Together, these results suggest that PLM‐derived representations are sensitive to stability changes caused by amino acid substitutions and that integrating PLM‐derived sequence information with structure‐based Δ*G* prediction frameworks may lead to a more robust and sensitive Δ*G* prediction tool.

Based on this idea, we adopt a complementary strategy in which structural representations provide coarse cues for Δ*G* prediction, which are then refined using sequence‐based contextual information. To encode protein backbone structures, we employ ProteinMPNN (Dauparas et al., 2022), an amino acid sequence prediction model similar to ESM‐IF, taking advantage of its accessible intermediate representations. To encode amino acid sequences, we use Evolutionary Scale Modeling 2 (ESM2), a well‐established protein language model developed by Lin et al. (2023). These structural and sequence representations are subsequently integrated using multi‐head cross‐attention (Vaswani et al., 2017), a widely used framework for capturing relationships between different types of information, to achieve more precise Δ*G* prediction. By combining these components, we construct a new Δ*G* prediction model, termed RINAMI (Residue‐attributed Interpretable Neural network for predicting Absolute folding free energy by Merging structure and sequence Information). RINAMI is then trained and evaluated on datasets in which Δ*G* values were measured under well‐controlled and standard experimental conditions, typically near room temperature (~25°C) and neutral pH (~7), to ensure consistency between predictions and experimental measurements.

RINAMI differs from recent machine‐learning‐based protein stability predictors in its prediction target, representation strategy, and mechanism for integrating sequence and structural information. ESM‐IF‐based approaches primarily score the compatibility between an amino acid sequence and a specified backbone conformation, whereas RINAMI is trained to predict the absolute folding free energy, Δ*G*, by jointly leveraging sequence‐derived and structure‐derived representations. In addition, many models based on PLMs or structure‐aware PLMs, as well as those incorporating thermodynamic consistency constraints, are designed to predict mutation‐induced stability changes, ΔΔ*G*, rather than absolute folding free energy, Δ*G* (Barducci et al., 2025; Rodrigues et al., 2018; Savojardo et al., 2025; Su et al., 2023). Although such methods provide valuable estimates of mutation‐induced stability effects, they are not directly optimized to estimate the thermodynamic stability of a target folded state relative to the unfolded ensemble. In contrast, RINAMI uses multi‐head cross‐attention to couple ESM2‐derived sequence representations with ProteinMPNN‐derived structural representations, allowing structural features to be reweighted in a sequence‐dependent manner. This architecture also supports residue‐level interpretation through a residue‐amino‐acid‐wise Δ*G* matrix that resolves contributions across residue positions and amino acid identities, as detailed in Section 2.1. To evaluate the effectiveness of this integrated sequence‐structure strategy, we benchmarked RINAMI on datasets comprising both natural and designed proteins measured under comparable standardized conditions. In these benchmarks, RINAMI outperformed the tested stability indicators and prediction methods, such as Rosetta energy scores (Simons et al., 1997), which are widely used computational metrics for estimating protein structural stability, as well as Cagiada's Δ*G* prediction method. Moreover, ablation analysis demonstrates that integrating sequence and structural representations contributes to the performance improvements.

## 2 RESULTS

### 2.1 Architecture and training of RINAMI

We constructed the RINAMI architecture to predict Δ*G* by separately encoding amino acid sequence information and three‐dimensional structural information, and subsequently integrating these representations via multi‐head cross‐attention. The detailed procedure is described below.

**FIGURE 1:**
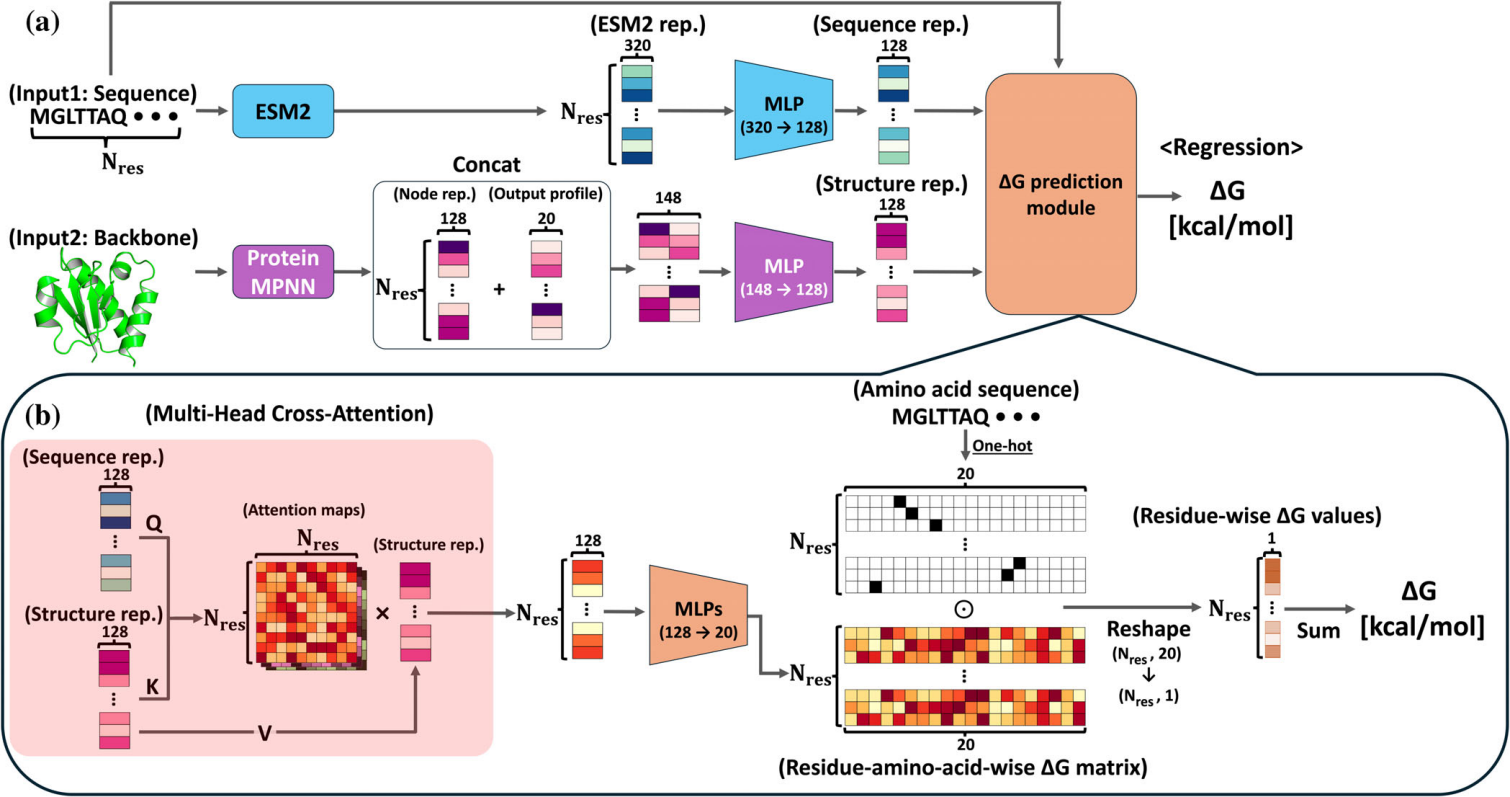
Schematic illustration of the RINAMI architecture. (a) Overview of the RINAMI model architecture. The amino acid sequence is first embedded using Evolutionary Scale Modeling 2 (ESM2) to obtain ESM2 representations. In parallel, the backbone structure is processed by ProteinMPNN, a protein sequence‐design model based on message‐passing neural networks, to generate the node representations and output probability profiles, which are concatenated to form the structure representation. The sequence‐derived and structure‐derived representations are then independently transformed by multi‐layer perceptrons (MLPs) and passed to the Δ*G* prediction module to estimate the absolute folding free energy (Δ*G*). (b) Detailed illustration of the Δ*G* prediction module. The structural representation is reweighted through multi‐head cross‐attention, where the amino acid representation serves as the query (*Q*) and the structural representation provides the key (*K*), and value (*V*) vectors. The resulting reweighted representations are converted by MLPs into a residue‐amino‐acid‐wise Δ*G* matrix. For each residue, the partial Δ*G* score corresponding to its original amino acid is selected and summed across positions to yield the final predicted Δ*G* value.

First, as shown in Figure 1a, the amino acid sequence is provided as input to ESM2, whereas the protein backbone structure is provided to ProteinMPNN. From these two models, we extracted three types of feature representations. The ESM2 representation captures context‐dependent features of each amino acid residue encoded by the pretrained ESM2 model from sequence information and consists of a sequence of 320‐dimensional vectors, one for each residue. The ProteinMPNN node representation captures the local structural environment surrounding each residue within the three‐dimensional protein structure and is represented as a sequence of 128‐dimensional vectors. In addition, the ProteinMPNN output profile represents the predicted probability distribution over the 20 amino acids at each residue position and is expressed as a sequence of 20‐dimensional vectors. These two ProteinMPNN‐derived features are then concatenated to form a unified structure‐based representation. The ESM2 representation and the concatenated structure‐based representation are then independently processed through separate multi‐layer perceptrons (MLPs) to generate refined sequence‐based and structure‐based representations, which are passed to the multi‐head cross‐attention module as illustrated in Figure 1b and mentioned below.

Second, the sequence‐based and structure‐based representations are integrated with a multi‐head cross‐attention mechanism, in which the sequence representation serves as the query (Q), while the structural representation serves as both the key (K) and the value (V) (Figure 1b). This design enables the model to reweight the structural representation according to sequence context. The choice of the structural representation as the value (*V*) is motivated by prior studies showing that coarse Δ*G* estimates can be obtained from structural information alone, as demonstrated by Cagiada et al., whereas further refinement can be achieved by incorporating sequence information.

The resulting reweighted structural representation is then passed through additional MLPs to generate a residue‐amino‐acid‐wise Δ*G* matrix, in which each element corresponds to the partial Δ*G* score of a specific amino acid at a given residue position, as shown in Figures 1b and 4a. The predicted protein‐level Δ*G* is obtained by summing the partial Δ*G* scores corresponding to the amino acids in the input sequence. Notably, this matrix‐based formulation enables interpretation of the contributions of individual residues and amino acids to the predicted Δ*G*, as discussed in detail in Section 2.4.

We trained RINAMI using the Mega‐scale protein stability dataset curated by Tsuboyama et al. (2023), which contains experimentally measured Δ*G* values for several hundred small protein domains and their sequence variants. The Δ*G* values were measured under controlled experimental conditions, typically at room temperature (~25°C) and near‐neutral pH (~7), providing a consistent basis for model training. For model development and evaluation, we generated three independent training/validation/test splits of the dataset at an 8:1:1 ratio using a clustering‐based partitioning strategy to minimize data leakage (see Section 4.1). The number of examples in each subdataset of each split is shown in Table S1. For each split, RINAMI was trained three times with different random initializations to assess the robustness of model training and predictive performance. After training, we selected the model parameters to be used for downstream evaluation from the saved epochs of each run according to validation‐set performance. Specifically, we chose the epoch with the highest sum of Pearson's correlation coefficient (Pearson's *R*) and Spearman's rank correlation coefficient (Spearman's *R*) between predicted and experimentally measured Δ*G* values. Test‐set performance for all splits and random initializations is reported in Table S2.

### 2.2 Evaluation of RINAMI's Δ *G* prediction accuracy and comparison with existing methods

Before evaluating RINAMI's predictive performance, we selected, for each split, the lowest‐performing model from among three models trained with different parameter initializations as the representative model for comparison with existing methods, thereby ensuring a conservative assessment of RINAMI's performance. To objectively identify the lowest‐performing run within each split, we used four evaluation metrics: root mean squared error (RMSE), mean absolute error (MAE), Pearson's *R*, and Spearman's *R*. Each metric was first scaled from 0 to 1, with 0 corresponding to the best value and 1 corresponding to the worst value within that split. The normalization was performed according to the equation described in Section 4.4. We then calculated the average of the four normalized scores, which is reported as the “Average Normalized Metric Score” in Table S2. The run with the highest Average Normalized Metric Score was selected as the lowest‐performing run.

To evaluate the accuracy of RINAMI for absolute Δ*G* prediction, we used two benchmark datasets. The first benchmark consisted of the test subdatasets from the three independent Mega‐scale dataset splits described above, comprising approximately 66,000 small protein domains and sequence variants with lengths of 40–72 residues. Detailed sample counts for the test subdataset of each split are provided in Table S1. The second benchmark was the thermal stability dataset curated by Maxwell et al. (2005) (hereafter, the Maxwell dataset), which contains 22 small‐ to medium‐sized natural proteins of 65–151 residues and one larger protein of 338 residues. All benchmark Δ*G* measurements were obtained under conditions comparable to those used for training, typically at approximately 25°C and near‐neutral pH, thereby reducing variation attributable to experimental conditions and enabling direct comparison between predicted and measured Δ*G* values (see Sections 4.1 and 4.2). No entries in the benchmark test sets were included in the corresponding training subdatasets, and the external Maxwell dataset was not used for model training. These benchmarks therefore provide an assessment of RINAMI's predictive performance while minimizing potential data leakage. For the Mega‐scale benchmark, we compared RINAMI with existing methods using evaluation metrics averaged across the test subdatasets from the three independent splits. For the Maxwell benchmark, the final RINAMI prediction was defined as the average of the predictions from the three conservatively selected RINAMI models, one from each independent split. This averaging strategy was used because the Maxwell dataset was independent of the Mega‐scale training subdatasets and therefore served as a common external benchmark for all three selected models. Averaging predictions across independently trained models was used to reduce split‐ and initialization‐dependent variability and to obtain a more stable estimate of RINAMI's generalization performance on this external dataset.

**FIGURE 2:**
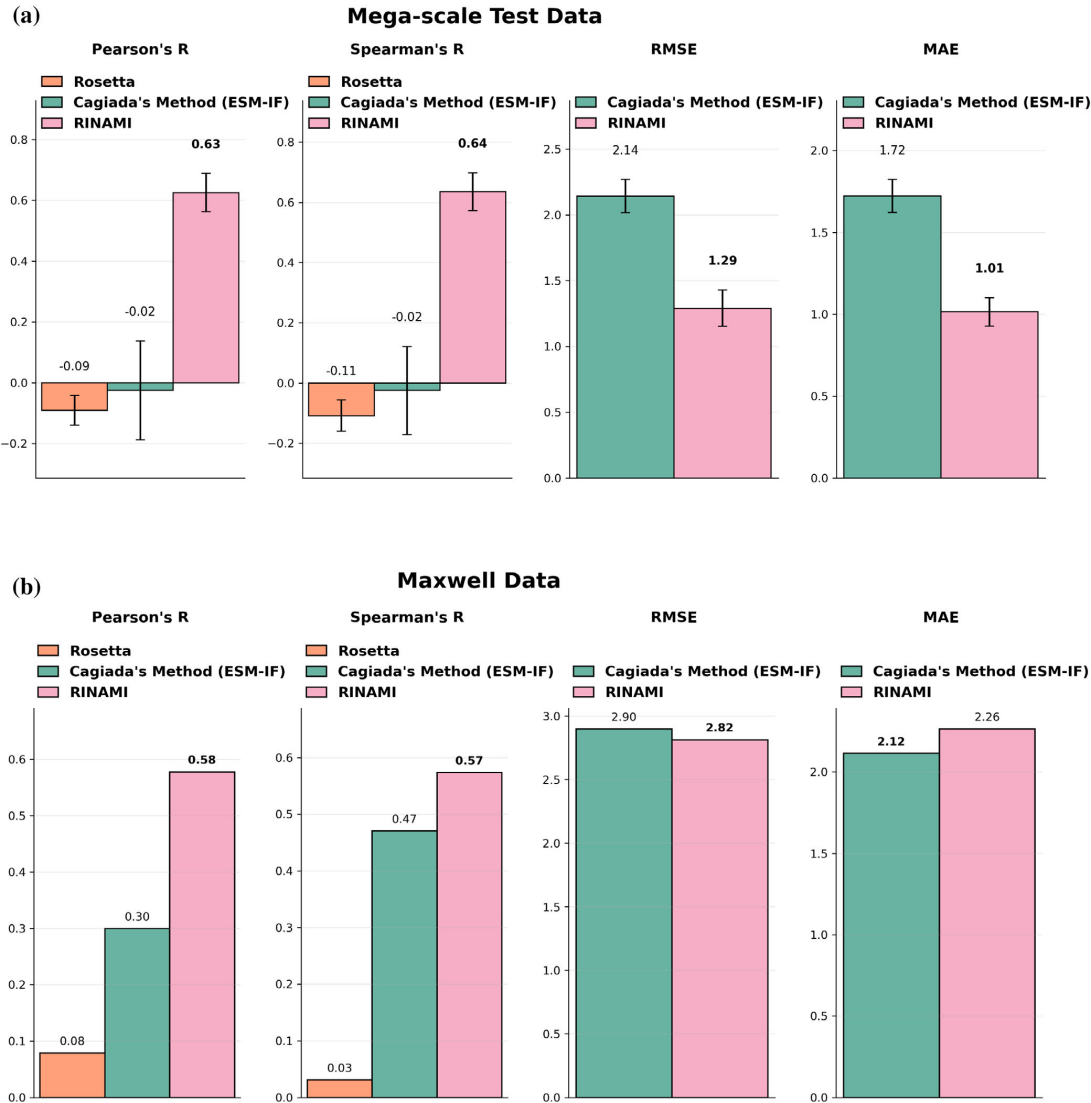
Benchmark comparison of Δ*G* prediction accuracy. (a) Comparison of the Δ*G* prediction performance among the tested existing methods and RINAMI on the Mega‐scale test subdatasets. For each evaluation metric, the mean value is shown with error bars representing the standard deviation across the three cluster‐based data splits. (b) Comparison of the Δ*G* prediction performance among the tested existing methods and RINAMI on the Maxwell test dataset. Prediction performance was evaluated using Pearson's correlation coefficient (Pearson's *R*), Spearman's rank correlation coefficient (Spearman's *R*), root mean squared error (RMSE), and mean absolute error (MAE). RMSE and MAE are reported only for Cagiada's method and RINAMI because the Rosetta energy score is not expressed in kcal/mol.

Across both benchmarks, RINAMI showed improved or comparable performance over existing methods for absolute Δ*G* prediction. We compared RINAMI with two representative stability estimation approaches: Rosetta energy scores, which are based on an energy function combining molecular mechanics terms with statistical potentials derived from structural databases, and the ESM‐IF‐based Δ*G* prediction method proposed by Cagiada et al. Across the benchmark evaluations, RINAMI achieved the highest Pearson's *R* and Spearman's *R*, as well as the lowest RMSE, relative to experimentally measured Δ*G* values. RINAMI also showed lower MAE than the method of Cagiada et al. on the Mega‐scale test subdatasets and comparable MAE on the Maxwell dataset (Figure 2). Taken together, these results indicate that RINAMI provides improved absolute Δ*G* prediction relative to the tested existing approaches, particularly for the small‐ and medium‐sized proteins represented in these benchmarks.

Because the reliability of performance estimates can depend on benchmark size, we additionally performed bootstrap analyses to quantify the uncertainty of the evaluation metrics for each method on both the Mega‐scale and Maxwell benchmarks. For the Mega‐scale test subdatasets, the 95% confidence intervals were narrow and the bootstrap standard deviations were small across all three splits, reflecting the large number of evaluated entries (Table S3). In these analyses, RINAMI consistently showed higher Pearson's *R* and Spearman's *R* than Rosetta energy scores and Cagiada et al.'s method, as well as lower RMSE and MAE than Cagiada et al.'s method. The confidence intervals generally showed little or no overlap between RINAMI and the existing methods, further supporting the robustness of RINAMI's improved predictive performance on the Mega‐scale benchmark. In contrast, the Maxwell dataset contained only a limited number of proteins, resulting in substantially wider confidence intervals for all methods (Table S4). On this external benchmark, RINAMI showed higher Pearson's *R* and Spearman's *R* than the tested methods, whereas the error‐based metrics showed larger uncertainty and were comparable to those of Cagiada et al.'s method. Thus, although RINAMI showed improved correlation‐based performance and comparable error‐based performance on this external benchmark, the uncertainty associated with the small Maxwell dataset indicates that broader generalizability should be further evaluated using larger and more diverse external datasets.

### 2.3 The combination of the amino acid representation with the structural representation improves the predictive accuracy

To evaluate the benefit of integrating amino acid and structural representations, we conducted an ablation study. For this purpose, we constructed a baseline model for comparison with RINAMI, the architecture of which is illustrated in Figure S2. This baseline removes sequence‐structure interaction by replacing the multi‐head cross‐attention with an MLP that processes only the structural representation for a given input protein. We then trained this baseline model using the same optimizer, learning‐rate schedule, and batch size as those used for RINAMI (see Section 4.6) to ensure that any observed differences arise from architectural differences rather than from training conditions. Finally, we assessed and compared the predictive performance of the baseline model and RINAMI on both the Mega‐scale test subdatasets and the Maxwell dataset.

**FIGURE 3:**
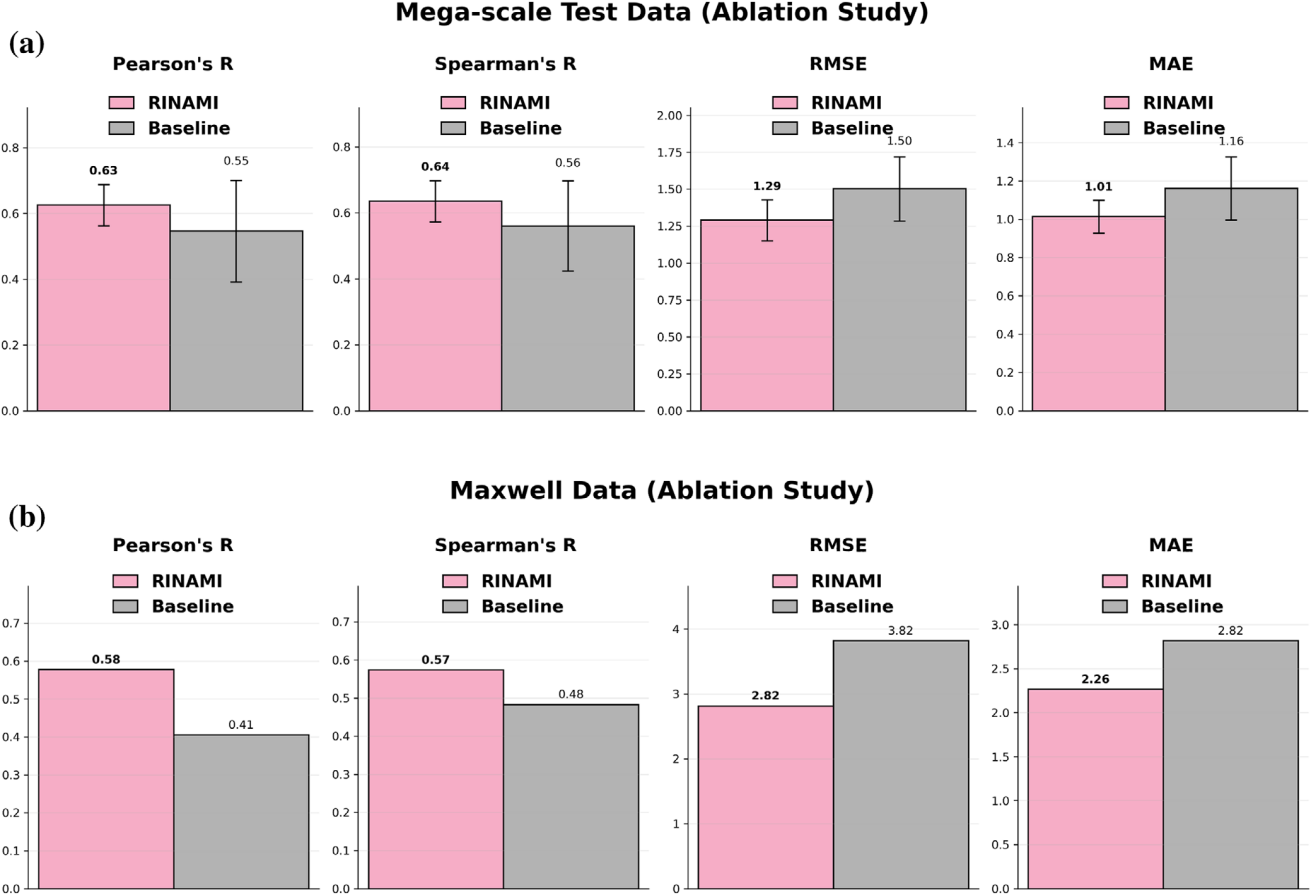
Ablation study demonstrating the benefit of integrating sequence and structural representations for Δ*G* prediction. (a) Comparison of the Δ*G* prediction performance between the baseline model and RINAMI on the Mega‐scale test subdatasets. For each evaluation metric, the mean value is shown with error bars representing the standard deviation across the three cluster‐based data splits. (b) Comparison of the Δ*G* prediction performance between the baseline model and RINAMI on the Maxwell test dataset. Prediction performance was evaluated using Pearson's correlation coefficient (Pearson's *R*), Spearman's rank correlation coefficient (Spearman's *R*), root mean squared error (RMSE), and mean absolute error (MAE).

Across both the Mega‐scale test subdatasets and the Maxwell dataset, RINAMI consistently outperformed the baseline model in Δ*G* prediction. As shown in Figure 3, in both evaluations, RINAMI achieved higher Pearson's *R* and Spearman's *R* values between predicted and experimentally measured Δ*G* values, as well as lower RMSE and MAE values, than the baseline model. These results demonstrate that RINAMI achieved improved qualitative predictive performance, as evidenced by higher Pearson's *R* and Spearman's *R* reflecting stronger agreement with experimental trends. Lower RMSE and MAE values indicate enhanced quantitative accuracy in predicting experimental Δ*G* values. Furthermore, Wilcoxon signed‐rank tests comparing the absolute prediction errors of RINAMI and the baseline model showed highly significant performance differences across the three Mega‐scale test subdatasets, with *p*‐values below 0.01 in all splits (*p* = 5.95 × 10^−145^, *p* < 1.00 × 10^−300^, and *p* = 1.39 × 10^−25^). In the Maxwell dataset, which contained only 23 proteins, RINAMI showed a lower MAE than the baseline model, but the reduction in absolute prediction errors was not statistically significant (*p* = 1.00 × 10^−1^). Thus, the statistically significant improvements observed across the Mega‐scale test subdatasets support the effectiveness of sequence–structure integration in the large‐scale benchmark, whereas the ablation result on the small external Maxwell dataset should be interpreted cautiously because of its limited sample size. These findings indicate that reweighting structural representations through multi‐head cross‐attention with amino acid representations improves Δ*G* prediction in the Mega‐scale benchmark, while its effect on independent external benchmarks should be evaluated further using larger datasets.

### 2.4 RINAMI captures the physicochemical contributions of individual amino acids to protein stability

**FIGURE 4:**
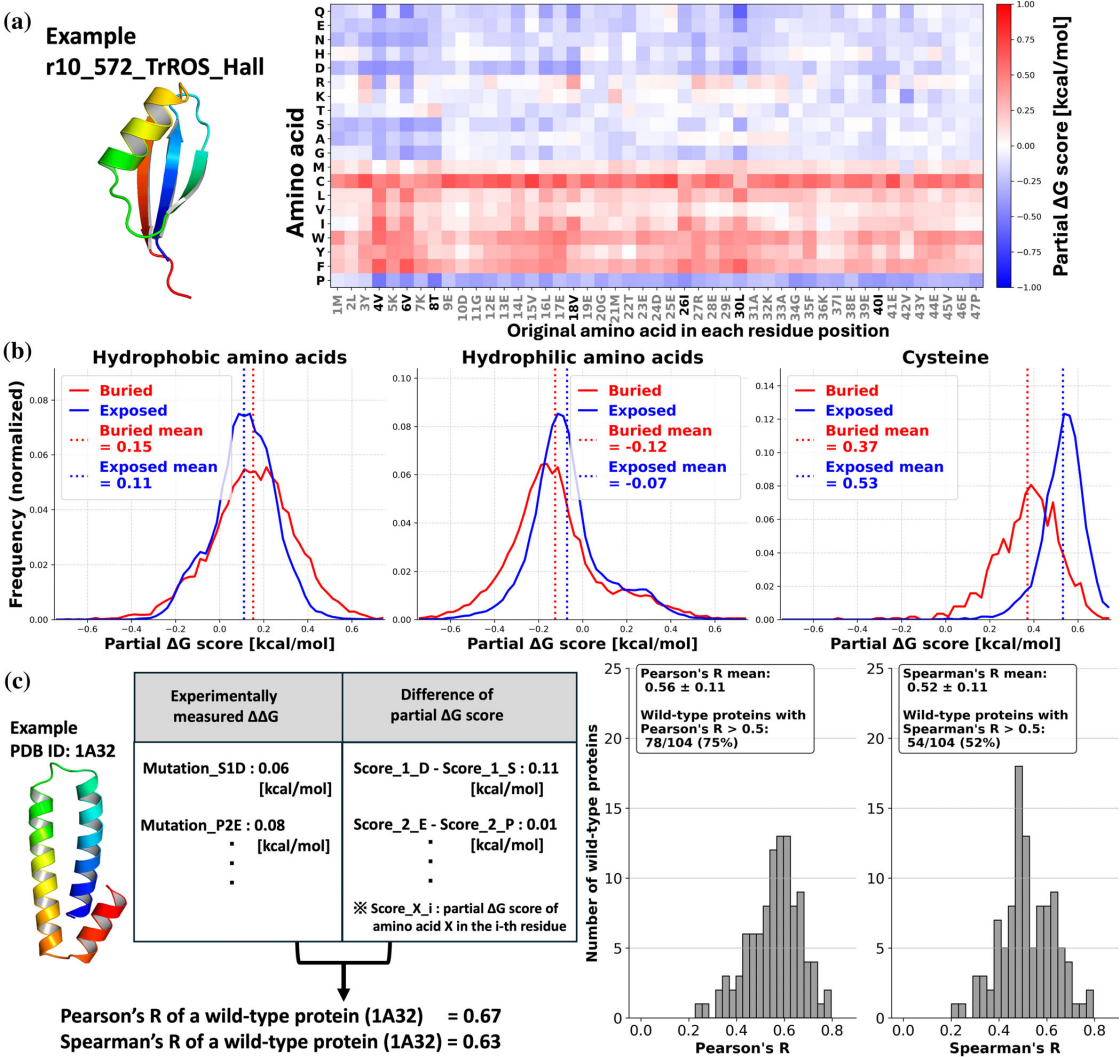
Residue–amino‐acid‐wise Δ*G* matrix and its validation using experimental single‐mutation ΔΔ*G* data. (a) Residue‐amino‐acid‐wise Δ*G* matrix of a representative wild‐type protein, r10_572_TrROS_Hall, in the Mega‐scale test subdataset. Each cell in the matrix is colored according to the predicted partial Δ*G* score, with higher partial Δ*G* scores shown in red and lower partial Δ*G* scores shown in blue. On the *x*‐axis, buried and exposed residues are highlighted in black and gray, respectively. The *y*‐axis indicates the 20 natural amino acids. (b) Distributions of predicted partial Δ*G* scores for each amino acid at each residue position. These distributions were generated from Δ*G* predictions for the wild‐type proteins in the Mega‐scale validation and test subdatasets. In each panel, the distributions of partial Δ*G* scores for buried and exposed residues are shown as red and blue solid lines, respectively, and their mean values are indicated by red and blue dotted vertical lines. (c) Distributions of Pearson's correlation coefficient (Pearson's *R*) and Spearman's rank correlation coefficient (Spearman's *R*) calculated separately for each wild‐type protein between experimentally measured ΔΔ*G* values and mutation‐associated differences in partial Δ*G* scores. The left schematic illustrates the calculation procedure for one representative wild‐type protein. For each wild‐type protein, single‐mutant variants derived from the same wild‐type protein were collected, and a table was constructed in which each row corresponds to one single mutation and contains the experimentally measured ΔΔ*G* value and the corresponding difference in partial Δ*G* score. Pearson's *R* and Spearman's *R* were then calculated across the single‐mutant variants belonging to that wild‐type protein.

Beyond predictive accuracy, we examined whether RINAMI captures physicochemical determinants of stability. To investigate the key factors influencing Δ*G* estimation, we analyzed residue‐amino‐acid‐wise Δ*G* matrices generated by RINAMI for wild‐type proteins in the Mega‐scale validation and test subdatasets of splits 1, 2, and 3, containing 28, 42, and 34 wild‐type proteins, respectively (104 proteins in total). Here, we refer to wild‐type proteins as cluster‐representative proteins selected during the data splitting described in Section 4.1 that are included in the validation and test subdatasets, annotated as *mut_type* = “wt” in the Mega‐scale dataset, and associated with continuous Δ*G* labels. This definition includes both naturally occurring and computationally designed proteins. Each cell in these matrices represents a partial Δ*G* score that reflects the compatibility between the physicochemical properties of a given amino acid and the local structural environment at its corresponding residue position (Figure 4a).

Prior to the analysis of these matrices, partial Δ*G* scores were grouped into six categories defined by the combination of residue exposure (buried or exposed) and three amino acid types described below to isolate physicochemical effects. This grouping was performed through the following two classification steps. First, we classified all residues within the 104 wild‐type proteins in the Mega‐scale validation and test subdatasets of each split into the two classes: buried residues and solvent‐exposed residues (see Section 4.8). This classification accounts for the structural context of each residue. Second, we classified the 20 standard amino acids into three categories: **hydrophobic**, **hydrophilic**, and **cysteine** to consider the effects of amino acids based on their physicochemical properties. This classification was based on the following criteria:

- **Cysteine** has unique properties due to its ability to form disulfide bonds.
- **Hydrophobic amino acids** (alanine, phenylalanine, isoleucine, leucine, methionine, and valine) were defined as those with positive hydropathy indices (Kyte & Doolittle, 1982) among the remaining amino acids.
- **Hydrophilic amino acids** (aspartic acid, glutamic acid, glycine, histidine, lysine, asparagine, proline, glutamine, arginine, serine, threonine, tyrosine, and tryptophan) were defined as those with negative hydropathy indices (Kyte & Doolittle, 1982) among the remaining amino acids.

After these preparatory steps, the analysis indicated that RINAMI accounts for the physicochemical properties of each amino acid in each residue position. This conclusion is supported by the following analyses. First, we analyzed the distribution of the partial Δ*G* scores within each category (Figure 4b). Second, we examined the individual residue‐amino‐acid‐wise Δ*G* matrices (Figure 4a). Third, we investigated correlations between experimentally measured ΔΔ*G* of deep mutation scanning and the difference of partial Δ*G* scores of the original amino acid and other amino acids, for each wild‐type protein (Figure 4c). These three analyses capture global, residue‐level, and protein‐level patterns in the Δ*G* predictions made by RINAMI, respectively. Together, they revealed that RINAMI successfully captures three key physicochemical effects relevant to protein structural stability and reflects the mutational effects for each protein:

1. Buried hydrophilic residues tend to show more negative Δ*G* scores than exposed hydrophilic residues, indicating a destabilizing effect. This global trend is apparent in the second panel from the left of Figure 4b. At the individual residue level, Figure 4a further supports this observation, showing that partial Δ*G* scores for buried hydrophilic amino acids are consistently low, in agreement with their known destabilizing effect. For instance, at most buried positions in the structure of a de novo designed protein (r10_572_TrROS_Hall; residues 4, 6, 18, 30, and 40), hydrophilic amino acids exhibit lower partial Δ*G* scores than when they are located in the other exposed positions.
2. Buried hydrophobic residues, in contrast, display more positive Δ*G* scores than their exposed counterparts (the leftmost panel of Figure 4b). This tendency reflects their stabilizing role in the structural cores of proteins, although the difference between exposed and buried environments is modest.
3. Cysteine residues consistently show high positive Δ*G* scores regardless of solvent exposure (the rightmost panel of Figure 4b), aligning with their known dual role: contributing to the hydrophobic core (Nagano et al., 1999) and forming disulfide bonds. Notably, the rightmost panel of Figure 4b shows that exposed cysteines contribute more to Δ*G* than buried ones, likely due to the greater free energy gain from disulfide bond formation (Cheek et al., 2006) compared to hydrophobic interactions.
4. For more than half of the wild‐type proteins, clear correlations (Pearson's *R*, Spearman's *R* > 0.5) were observed between the experimentally measured ΔΔ*G* values and the differences in partial Δ*G* scores corresponding to each single mutation (Figure 4c). These results indicate that RINAMI captures mutation‐induced stability changes in individual proteins. These protein‐level correlations were generally consistent across all splits and training runs (Figure S3). We further evaluated the robustness of the residue‐amino‐acid‐wise Δ*G* matrices across independent random initializations. Because these matrices are protein‐specific, a fair direct comparison of the explanatory outputs requires the same wild‐type protein to be evaluated across models. In the present cluster‐based 8:1:1 train/validation/test partitions, however, the validation and test subdatasets contained different wild‐type proteins across different splits. Therefore, we quantified the robustness of these explanatory outputs across independent training runs within each split. The residue‐amino‐acid‐wise Δ*G* matrices were highly similar across independently initialized training runs, as quantified by Pearson's *R* and Spearman's *R* (Figure S4). These results indicate that the residue‐level explanatory patterns captured by RINAMI were reproducible and not specific to a single random initialization.

These analyses indicate that the Δ*G* values predicted by RINAMI are interpretable in terms of amino‐acid physicochemical properties, which the model appears to capture across a wide range of structural and sequence contexts. This sensitivity to residue‐specific physicochemical properties prompted us to ask whether RINAMI could also estimate mutation‐induced changes in stability, despite being trained to predict absolute Δ*G* rather than ΔΔ*G*. To test this, we calculated mutation‐induced ΔΔ*G* values as the difference between the RINAMI‐predicted Δ*G* values for each mutant sequence and its corresponding wild‐type sequence. We then compared these RINAMI‐derived ΔΔ*G* values with experimentally measured ΔΔ*G* values. RINAMI‐derived ΔΔ*G* values showed substantial agreement with the experimental ΔΔ*G* values, indicating that the model captures stability effects associated with single amino acid substitutions. As shown in Figure S5, for most of the 104 wild‐type proteins, the RINAMI‐predicted ΔΔ*G* values for single amino acid substitutions exhibited Pearson's or Spearman's correlation coefficients greater than 0.5 with the experimentally measured ΔΔ*G* values.

### 2.5 RINAMI exhibits lower performance for large proteins compared with small proteins

**FIGURE 5:**
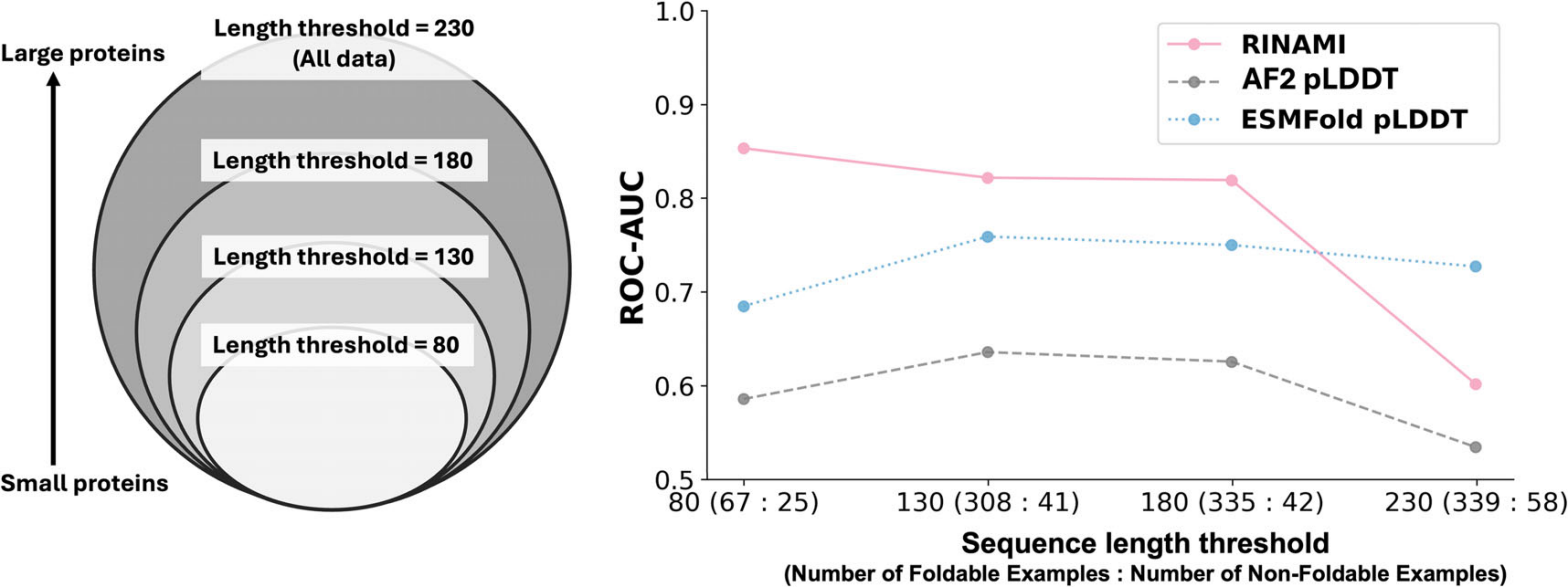
Benchmark evaluation of foldability prediction across protein length thresholds. Left: Schematic overview of the stratification of the benchmark dataset by sequence length threshold. Right: Comparison of foldability prediction performance among RINAMI, AlphaFold2 predicted local distance difference test (AF2 pLDDT), and ESMFold pLDDT. The *x*‐axis indicates the sequence length threshold used to stratify the dataset, together with the number of foldable and non‐foldable proteins included in each subdataset. The *y*‐axis indicates the receiver operating characteristic‐area under the curve (ROC‐AUC) value for foldability prediction in each subdataset.

Next, we examined the protein size range over which RINAMI maintains predictive accuracy. Although Section 2.2 demonstrates improved Δ*G* prediction by direct regression for small‐ and medium‐sized proteins, an equivalent assessment for large proteins is currently not feasible because suitable experimentally measured Δ*G* datasets for large proteins, obtained under standardized conditions, are not available. To approximate performance in this regime, we assessed foldability, defined here as the ability of a protein to uniquely adopt a stable target structure, using a benchmark set of de novo designed proteins with sequence lengths ranging from 58 to 193 residues. For this benchmark test, we utilized the dataset curated by Garcia et al., which provides structural predictions by AF2 (Jumper et al., 2021) and ESMFold (Lin et al., 2023) together with experimentally validated information on protein expression, secondary structure formation, and monomericity (Garcia et al., 2025). From this resource, we extracted entries with experimentally validated secondary structure formation to construct the Garcia dataset and stratified this dataset into subdatasets by amino acid sequence length (Figure 5; see Section 4.3). Entries forming stable intended folds were labeled as “foldable,” whereas entries not adopting the intended folds were labeled as “non‐foldable.” In this foldability prediction task, proteins with positive predicted Δ*G* values are classified as foldable, whereas those with negative predicted Δ*G* values are classified as non‐foldable.

We then assessed the performance of RINAMI in predicting foldability. In this prediction task, the Δ*G* value for each protein in the Garcia dataset is first predicted. These values are then passed through a sigmoid function to obtain normalized scores in the range [0, 1]. The resulting scores are interpreted as the probability that the corresponding protein folds into a unique, stable structure (see Figure S6). For foldability prediction, we used the mean Δ*G* prediction from the three conservatively selected RINAMI models, one from each independent split. This averaging strategy was used because the Garcia dataset was treated as an external benchmark independent of the Mega‐scale training subdatasets, analogous to the Maxwell dataset, and was intended to reduce split‐dependent variability.

This assessment indicated that RINAMI can predict foldability for small‐ and medium‐sized proteins, whereas its foldability prediction performance is limited for large proteins. As shown in Figure 5, RINAMI outperformed the pLDDT scores output by AF2 and ESMFold, which are widely used to assess the reliability of protein designs, particularly for small‐ and medium‐sized proteins. However, its predictive accuracy declined for the longest proteins in this benchmark, particularly those exceeding 180 residues.

This limitation likely arises from a strong bias of the training data toward small proteins, resulting in limited generalization to larger sequences. Although the Garcia dataset did not include proteins longer than 200 residues, the observed decline in performance for proteins exceeding approximately 180 residues suggests that RINAMI's application to proteins in or beyond this size range should be interpreted with caution. These findings underscore the importance of diversifying the training dataset—particularly by incorporating more experimentally validated data from large proteins—to further improve the practical utility and generalizability of RINAMI as a Δ*G* and foldability predictor. Because multi‐domain proteins may involve domain‐domain interactions, linker flexibility, and cooperative folding effects that are underrepresented in the current small‐protein‐biased training dataset, application to multi‐domain systems should also be regarded as exploratory until appropriate validation datasets become available.

## 3 DISCUSSION AND CONCLUSIONS

In this study, we developed RINAMI, a machine‐learning model that predicts folding free energy (Δ*G*) by integrating both sequence and structural information. Through benchmarking on the Mega‐scale and Maxwell datasets, RINAMI showed improved predictive accuracy compared with the tested existing approaches, including Cagiada et al.'s Δ*G* predictor and Rosetta energy scores. By integrating sequence and structural information, RINAMI provides a promising framework for Δ*G* prediction in small‐ and medium‐sized proteins, a task that remains challenging for PLM‐based approaches alone. In terms of computational cost, however, RINAMI required a longer inference time than the ESM‐IF‐based method proposed by Cagiada et al.; under our computational setting, the inference times for the 23 proteins in the Maxwell dataset were 21.86 s for RINAMI and 14.70 s for Cagiada et al.'s method, indicating that RINAMI was approximately 1.5 times slower. This increased computational cost may reflect the additional processing required to integrate both sequence‐ and structure‐derived representations. Thus, RINAMI involves a modest increase in inference time, but this cost is accompanied by improved Δ*G* prediction accuracy compared with the tested existing approaches.

Ablation experiments further demonstrated the importance of incorporating amino acid representations via multi‐head cross‐attention, which enhances predictive accuracy especially for the small‐sized proteins by reweighting structural representations in a sequence‐aware manner. In addition, residue‐level analyses revealed that RINAMI captures key physicochemical effects governing protein stability, such as the destabilizing effect of buried hydrophilic residues, the stabilizing contribution of buried hydrophobic residues, and the distinctive behavior of cysteine residues, particularly with respect to disulfide bond formation.

Despite these strengths, RINAMI showed reduced performance for large proteins, typically exceeding 180 residues, likely due to the bias of the available training data toward small proteins. Foldability prediction experiments further supported this size‐dependent limitation. Addressing this issue will require incorporating more experimentally validated Δ*G* data for large proteins in future training datasets. Therefore, the current version of RINAMI should be regarded as primarily validated for small‐ and medium‐sized proteins. Its application to proteins larger than approximately 180 residues or to multi‐domain proteins should be interpreted with caution until larger experimentally validated Δ*G* datasets become available.

Overcoming these limitations and developing a more generalizable Δ*G* prediction model will require two major advances. First, more accurate structural prediction methods are needed to capture subtle mutation‐induced structural differences. In the present study, we used ESMFold‐predicted structures for both training and inference of RINAMI. This strategy enables Δ*G* prediction for hundreds of thousands of protein variants without requiring experimentally determined structures, but it also introduces the risk that errors in predicted structures may propagate into ΔG prediction through the ProteinMPNN‐derived structural representations. Indeed, our additional analysis showed that when structural representations were shuffled among variants derived from the same wild‐type protein, the Δ*G* prediction accuracy substantially decreased, even though these variants shared high sequence similarity and were expected to have only minor structural differences (Figure S7). This result suggests that even subtle inaccuracies in predicted structures can cause appreciable errors in Δ*G* prediction. Although ESMFold provides accurate single‐sequence structure prediction and is sensitive to mutation‐induced structural changes compared with other currently available methods, its predictions are still not perfect (Feldman et al., 2026). Therefore, further improvement of structure prediction from a single amino acid sequence will be essential for enhancing the accuracy of Δ*G* prediction. Second, hybrid approaches that integrate machine learning with physics‐based energy potentials and simulation‐derived information will be important for further improving Δ*G* prediction. In this study, RINAMI improved Δ*G* prediction for small‐ and medium‐sized proteins by combining sequence‐derived and structure‐derived representations within an ML‐based framework. At the same time, however, its generalizability remained limited, partly because the available training data were biased toward small proteins and because the current model relies on a single predicted structure as input. Physics‐based and ensemble‐based information may help complement these limitations by capturing structural fluctuations, local flexibility, and energetic features that are difficult to infer from static structures alone. For example, Marcos et al. showed that long MD simulations can reveal dynamic fingerprints associated with protein thermostability (Marcos et al., 2012). DynaMut demonstrated that normal‐mode‐derived protein dynamics and graph‐based signatures can be integrated to predict mutation‐induced changes in protein conformation, flexibility, and stability (Rodrigues et al., 2018). Gill and McCully showed that MD simulations of de novo designed proteins can reproduce relative experimental stabilities and help identify stabilizing mutations (Gill & McCully, 2020). More recently, Bhagat and Padhi used multiscale simulations, energetic profiling, and rational redesign to analyze structural ensembles, conformational fluctuations, and physicochemical interactions in *Thermococcus* AMP phosphorylase and its DPBB domains, thereby providing a detailed view of thermostability and mutation‐induced stability changes (Bhagat & Padhi, 2026). Although these approaches are not directly equivalent to high‐throughput absolute Δ*G* prediction, they highlight the importance of conformational ensembles, dynamics‐related energetic profiles, and physics‐based constraints for understanding protein stability. Future extensions of RINAMI may therefore benefit from incorporating computationally efficient approximations of such information, including ensemble‐level structural descriptors, normal‐mode‐derived features, or physics‐inspired energetic terms, to improve accuracy and interpretability across a broader range of proteins.

Because the current implementation of RINAMI uses a single input structure, it does not explicitly account for conformational heterogeneity or residue‐level flexibility. A promising future direction is therefore to integrate MD‐informed or ensemble‐based machine‐learning approaches into the RINAMI framework. Recent studies have shown that ML models trained on MD‐derived data can predict residue‐level fluctuations or generate conformational ensembles at substantially reduced computational cost. For example, Flexpert‐3D estimates residue‐level flexibility quantified by root‐mean‐square fluctuation (RMSF) and has been shown to reproduce MD‐derived flexibility profiles across diverse proteins (Kouba et al., 2025). Similarly, AlphaFlow generates protein conformational ensembles more efficiently than conventional MSA subsampling or MD simulations while capturing ensemble properties consistent with MD‐derived conformational distributions (Jing et al., 2024). Incorporating the outputs or internal representations of such MD‐informed ML models into RINAMI as additional structural features or auxiliary prediction targets may improve the robustness of Δ*G* prediction by allowing the model to account for dynamic properties that are not captured by a single static structure. Thus, future versions of RINAMI that incorporate dynamic features may provide an efficient proxy for incorporating dynamic information into large‐scale protein design screening.

In conclusion, although further refinement is still needed, RINAMI provides an improved and interpretable approach for Δ*G* prediction in small‐ and medium‐sized proteins, and serves as a valuable computational tool for protein design and stability evaluation.

## 4 METHODS

### 4.1 Splitting the mega‐scale dataset into the training subdataset, validation subdataset, and test subdataset

For the training, validation, and testing of RINAMI, we prepared three independent data splits derived from the Mega‐scale dataset to assess the reproducibility of both the convergence behavior of the training process and the model's predictive performance.

First, from “Dataset #2” of the Mega‐scale dataset curated by Tsuboyama et al. (2023) for the Δ*G* prediction task, we extracted entries labeled with experimentally measured continuous Δ*G* values, together with entries labeled as “Δ*G* > 5 kcal/mol” or “Δ*G* < −1 kcal/mol.” Among these entries, proteins annotated as *mut_type* = “wt,” which were treated as wild‐type proteins in the study by Tsuboyama et al., were used for sequence clustering. Clustering was conducted with MMseqs2 (Steinegger & Söding, 2017), and sequences were assigned to the same cluster if they shared at least 25% sequence identity and if the alignment covered at least 80% of each sequence being compared. All mutant entries were assigned to the same cluster as their corresponding wild‐type protein.

The resulting clusters were then randomly allocated to the training, validation, and test subdatasets so that the total numbers of entries with continuous Δ*G* labels were approximately in an 8:1:1 ratio. Because this allocation was performed at the cluster level, all variants belonging to the same cluster were assigned to a single subdataset, thereby preventing data leakage between the training, validation, and test data. This cluster‐level allocation procedure was repeated three times to generate three distinct data splits with different distributions of clusters across the training, validation, and test subdatasets.

Entries labeled as “Δ*G* > 5 kcal/mol” or “Δ*G* < −1 kcal/mol” were not used for Δ*G* regression because they do not provide continuous Δ*G* values. Instead, for the foldability prediction task described in Section 4.5, only those extreme‐label entries whose clusters were assigned to the training subdataset in each split were used as extreme data during training.

All subdatasets included only entries with experimentally determined Δ*G* values measured under standardized conditions of room temperature and pH 7.4.

### 4.2 Curation of the Maxwell test dataset

The Maxwell dataset was used as an external benchmark dataset. The original dataset comprises experimentally measured thermodynamic properties, including Δ*G*, for 30 distinct proteins (Maxwell et al., 2005). From these 30 proteins, we excluded Protein G because a Δ*G* value was not available. In addition, to avoid overlap with the Mega‐scale subdatasets, we excluded ADAh2, ProteinL, NTL9, Ubiquitin, Spectrin_SH3, and SrcSH3 because the Mega‐scale dataset contained proteins sharing more than 25% sequence identity with these Maxwell proteins, with the aligned region covering at least 80% of both sequences in each pairwise comparison. Sequence similarity was assessed using MMseqs2. After these sequence comparisons and exclusions, the remaining 23 proteins were used as the benchmark dataset. All Δ*G* values in the Maxwell dataset were experimentally determined under standardized conditions of room temperature and pH 7.0.

### 4.3 Construction of the Garcia benchmark dataset

To assess the predictive accuracy of RINAMI for protein foldability, we constructed a benchmark dataset based on the collection curated by Garcia et al. (Garcia et al., 2025). From the original set of 614 de novo designed proteins, we extracted 405 proteins for which foldability had been experimentally assessed using circular dichroism (CD) spectroscopy. Furthermore, to avoid data leakage, we excluded eight proteins (NF2‐01, NF2‐02, NF2‐03, NF2‐04, dcs_A_1, dcs_A_2, dcs_A_3, and dcs_A_4) from the extracted dataset because each had at least one pairwise alignment to a protein in the Mega‐scale dataset in which the aligned region covered at least 80% of both sequences and the sequence identity exceeded 25%. Sequence similarity was assessed using MMseqs2. Consequently, the number of examples included in the resulting benchmark dataset is 397.

### 4.4 Calculation of the normalized metrics

In Section 2.2, we normalized each evaluation metric across the three training runs within each split to objectively identify the lowest‐performing run. Because higher values indicate better performance for Pearson's *R* and Spearman's *R*, these metrics were converted into normalized metrics using the following equation:

$$\text{Normalized metric}=\frac{\max\left(x\right)-x}{\max\left(x\right)-\min\left(x\right)}$$

where *x* represents the value of the corresponding evaluation metric for a given training run within a given split, and max(*x*) and min(*x*) represent the maximum and minimum values of that metric, respectively, across the three training runs in the same split. In this transformation, the best value within the split is assigned a normalized metric value of 0, whereas the worst value is assigned a value of 1.

In contrast, lower values indicate better performance for RMSE and MAE. Therefore, these metrics were normalized using the following equation:

$$\text{Normalized metric}=\frac{x-\min\left(x\right)}{\max\left(x\right)-\min\left(x\right)}$$

The same definitions of *x*, max(*x*), and min(*x*) were used for RMSE and MAE. This transformation also assigns a value of 0 to the best metric value and a value of 1 to the worst metric value within each split. After calculating the normalized metrics for Pearson's *R*, Spearman's *R*, RMSE, and MAE, we averaged the four normalized values to obtain the Average Normalized Metric Score for each training run. The run with the highest Average Normalized Metric Score was defined as the lowest‐performing run within that split.

### 4.5 Structural prediction by ESMFold

The Mega‐scale dataset does not provide structural information for protein variants. Therefore, we predicted protein structures for all variants in the training, validation, and test subdatasets. For this purpose, we employed ESMFold, which enables computationally efficient and accurate single‐sequence protein structure prediction (Lin et al., 2023) and has been reported to be sensitive to mutations that induce structural changes (Feldman et al., 2026). We used four recycling iterations, corresponding to the default configuration of ESMFold.

### 4.6 Training and validation of RINAMI

The training procedure of RINAMI consisted of two distinct prediction tasks.

#### 4.6.1 Foldability prediction task

Although the default architecture of RINAMI is designed to predict Δ*G* values, we repurposed the model to estimate protein foldability in this task. Specifically, the predicted Δ*G* values are transformed by a sigmoid function to map them to the range [0, 1] and interpreted as the probability that a protein folds into a unique and stable structure (Figure S6). For training and validation, experimentally measured Δ*G* values were binarized such that values ≤0 were labeled as non‐foldable (0) and values >0 as foldable (1), following the definition used in this study. The loss was computed as the binary cross‐entropy (BCE) between the predicted foldability probabilities and the binary labels.

#### 4.6.2 ΔG regression task

In this task, RINAMI predicts continuous Δ*G* values using its regression architecture, as shown in Figure 1. During training and validation, the loss was calculated using the Huber loss between the predicted Δ*G* values and the experimentally determined Δ*G* values.

To improve the Δ*G* regression accuracy, the foldability prediction task was interleaved with the Δ*G* regression task as an auxiliary objective. This training scheme was motivated by a previous study by Kiperwasser and Ballesteros that showed that interleaving an auxiliary task with a main task improves the performance of the primary task (Kiperwasser & Ballesteros, 2018). Accordingly, we scheduled one epoch of the foldability prediction task followed by three consecutive epochs of the Δ*G* regression task, and this cycle was repeated throughout training. During training, we used a batch size of 256 and a cosine learning rate scheduler with warmup (“get_cosine_schedule_with_warmup”) as implemented in the Transformers library for Python. Learning rates were set to 0.0, 0.0, and 1e−4 for ESM2, ProteinMPNN, and the Δ*G* prediction module, respectively, where a learning rate of 0.0 indicates that the parameters of the corresponding module were frozen. As a result of the multi‐task training mentioned above, the performance of the trained models was improved, as shown in Figure S8.

Because the training subdataset was highly imbalanced toward foldable proteins, we augmented the training data with Mega‐scale entries carrying censored Δ*G* labels to increase the number of non‐foldable instances and applied class weighting when calculating the BCE loss. In the original training subdataset, approximately 90% of the samples correspond to foldable proteins with positive Δ*G* values, whereas only about 10% represent non‐foldable proteins with negative Δ*G* values. Such severe class imbalance can hinder effective model training and bias predictions toward the majority class. To mitigate this issue, we added entries from the Mega‐scale dataset labeled with “Δ*G* > 5 kcal/mol” or “Δ*G* < −1 kcal/mol.” Because these data fall outside the range in which Δ*G* measurements can be considered reliable quantitative values, they were labeled as “Δ*G* > 5 kcal/mol” or “Δ*G* < −1 kcal/mol” rather than assigned exact Δ*G* values. Therefore, they could not be used for the regression task. However, they could reasonably be regarded as foldable or non‐foldable examples, respectively, and were thus used for the foldability prediction task thereby increasing the number of non‐foldable examples.

In addition, when calculating the BCE loss for foldability prediction, we set the positive‐class weight to pos_weight = (Number of non‐foldable samples)/(Number of foldable samples), where foldable proteins were treated as the positive class. Under this setting, the relative contribution of foldable examples, which constituted the majority class, was reduced, thereby balancing the total contributions of foldable and non‐foldable examples to the loss.

### 4.7 Calculation of the Rosetta energy scores

Rosetta energy scores for wild‐type and mutant protein structures were calculated with PyRosetta (Chaudhury et al., 2010). For each protein in the dataset, we loaded the corresponding structure into PyRosetta. For each structure, we then evaluated the standard full‐atom *ref2015* score function (Alford et al., 2017) and obtained residue‐level energies via the PyRosetta *Energies* object. The residue‐level energies were subsequently summed to obtain a Rosetta energy score for each protein.

### 4.8 Identification of the buried residues and exposed residues in the protein structure

In Section 2.4, we analyzed the distribution of partial Δ*G* scores in relation to residue location (buried or exposed). In this analysis, buried residues were defined as those with a relative solvent accessibility (RSA) (Lee & Richards, 1971) of less than 16%, as RSA serves as a reliable indicator of residue exposure. The 16% threshold was chosen following Momen‐Roknabadi et al. (2008), who identified this value as optimal for distinguishing buried residues from exposed ones.

## AUTHOR CONTRIBUTIONS

**Naoki Tomita:** Methodology; software; data curation; investigation; validation; formal analysis; funding acquisition; visualization; writing – original draft; writing – review and editing. **George Chikenji:** Conceptualization; validation; supervision; funding acquisition; project administration; writing – review and editing; resources.

## FUNDING INFORMATION

This work was supported by JSPS KAKENHI 26H01464, 25 K09527, and 22H00406 to G.C., and 26KJ1320 to N.T.; JST SPRING, JPMJSP2125; and the THERS Make New Standards Program for the Next Generation Researchers to N.T.

## CONFLICT OF INTEREST STATEMENT

The authors declare no conflicts of interest.

## Supporting information


**Figure S1:** Performance of Cagiada's method in predicting ΔG for natural and variant proteins. The left panel shows prediction results for a dataset consisting of proteins labeled as *mut_type* = “wt” from the Mega‐scale dataset and natural proteins from the Maxwell dataset. The right panel shows prediction results for variant proteins from the Mega‐scale dataset. The *x*‐ and *y*‐axes indicate the experimentally measured and predicted Δ*G* values, respectively. In each panel, Pearson's correlation coefficient (Pearson's *R*) and Spearman's rank correlation coefficient (Spearman's *R*) between predicted and experimentally measured Δ*G* values are shown in the bottom‐right text box. The low Pearson's *R* for variant proteins indicates that Cagiada's method shows poor predictive performance for variant Δ*G* prediction, whereas the Pearson's *R* for proteins in the left panel indicates moderate agreement with experimental trends.
**Table S1:** Number of samples included in each subdataset. For each data split, the Mega‐scale dataset was partitioned using the clustering‐based method described in Section 4.1. “Extreme Positive/Negative Data” indicates entries labeled as “Δ*G* > 5 kcal/mol” or “Δ*G* < −1 kcal/mol,” which were used only for the foldability prediction task. “Numeric Data” indicates entries with numeric Δ*G* values, which were used for both the foldability prediction and Δ*G* regression tasks.
**Table S2:** Predictive performance of RINAMI across three independent cluster‐based data splits and three independent training runs per split. Predictive performance is quantified using Pearson's correlation coefficient (Pearson's *R*) and Spearman's rank correlation coefficient (Spearman's *R*), root mean squared error (RMSE), and mean absolute error (MAE) between predicted and experimentally measured Δ*G* values. The first nine rows report the performance on the Mega‐scale test subdataset for each split–run combination. For each split, RINAMI was trained three times using different random initializations. Mean values and standard deviations (Std) are shown for the models trained within each split, for all nine trained models, and for the lowest‐performing models selected from the three splits. The “Average Normalized Metric Score” is a composite score used to identify the lowest‐performing model; higher values indicate lower predictive performance. Details of its calculation are described in Section 2.2 and Section 4.4.
**Table S3:** Bootstrap analysis of predictive performance for each method on the test subdataset of each Mega‐scale dataset split. Predictive performance is quantified using Pearson's correlation coefficient (Pearson's *R*) and Spearman's rank correlation coefficient (Spearman's *R*), root mean squared error (RMSE), and mean absolute error (MAE) between predicted and experimentally measured Δ*G* values. For each analysis, 10,000 bootstrap iterations were performed. “Observed” indicates the metric value calculated using the full test subdataset before bootstrap resampling. “Bootstrap Mean” and “Bootstrap Std” indicate the mean and standard deviation of the metric values obtained from bootstrap resampling, respectively. “95%CI Low” and “95%CI High” indicate the lower and upper bounds of the 95% confidence interval, respectively. RMSE and MAE were not calculated for Rosetta energy scores because these scores are not expressed in (kcal/mol).
**Table S4:** Bootstrap analysis of predictive performance on the external Maxwell dataset across all methods. Predictive performance is quantified using Pearson's correlation coefficient (Pearson's *R*) and Spearman's rank correlation coefficient (Spearman's *R*), root mean squared error (RMSE), and mean absolute error (MAE) between predicted and experimentally measured Δ*G* values. For each analysis, 10,000 bootstrap iterations were performed. “Observed” indicates the metric value calculated using the full Maxwell dataset before bootstrap resampling. “Bootstrap Mean” and “Bootstrap Std” indicate the mean and standard deviation, respectively, of the metric values obtained from bootstrap resampling. “95%CI Low” and “95%CI High” indicate the lower and upper bounds, respectively, of the 95% confidence interval. RMSE and MAE were not calculated for Rosetta energy scores because these scores are not expressed in (kcal/mol).
**Figure S2:** Overview of the baseline model. In this model, the multi‐head cross‐attention module, which integrates the structural and sequence representations in RINAMI, is replaced with a multi‐layer perceptron (MLP) that processes only the structural representation. This baseline model was used to evaluate the contribution of sequence–structure integration to Δ*G* prediction.
**Figure S3:** Correlations between experimental single‐mutation ΔΔ*G* values and mutation‐associated differences in partial Δ*G* scores across data splits and training runs. (a) Distributions of Pearson's correlation coefficient (Pearson's *R*) values calculated separately for each wild‐type protein in the validation and test subdatasets of each split. These *R* values were calculated between experimentally measured ΔΔ*G* values and the corresponding differences in partial Δ*G* scores for each single mutation. (b) Distributions of Spearman's rank correlation coefficient (Spearman's *R*) values calculated separately for each wild‐type protein in the validation and test subdatasets of each split. These *R* values were calculated between experimentally measured ΔΔ*G* values and the corresponding differences in partial Δ*G* scores for each single mutation.
**Figure S4:** Similarity of residue–amino‐acid‐wise Δ*G* matrices across independent training runs for each wild‐type protein. For each wild‐type protein included in the validation and test subdatasets of each split, residue–amino‐acid‐wise Δ*G* matrices derived from three independent training runs within the same split were compared in an all‐versus‐all manner. Horizontal bars represent the mean Pearson's correlation coefficient (Pearson's *R*) and Spearman's rank correlation coefficient (Spearman's *R*) values across the three pairwise comparisons, and error bars represent the standard deviations across these pairwise comparisons.
**Figure S5:** Distributions of Pearson's correlation coefficient (Pearson's *R*) and Spearman's rank correlation coefficient (Spearman's *R*) values calculated separately for each wild‐type protein between experimentally measured ΔΔ*G* values and RINAMI‐predicted ΔΔ*G* values. The left schematic illustrates the calculation procedure for one representative wild‐type protein. For each wild‐type protein, single‐mutant variants derived from the same wild‐type protein were collected, and a table was constructed containing the experimentally measured ΔΔ*G* value and the RINAMI‐predicted ΔΔ*G* value for each mutation. The Pearson's *R* and Spearman's *R* values were then calculated across the single‐mutant variants belonging to that wild‐type protein. Experimental ΔΔ*G* values were calculated from the measured Δ*G* values of each single‐mutant variant and its corresponding wild‐type protein. RINAMI‐predicted ΔΔ*G* values were calculated from the predicted Δ*G* values of each single‐mutant variant and its corresponding wild‐type protein.
**Figure S6:** Overview of the foldability prediction task. Most of the prediction process is the same as that depicted in Figure 1. In this architecture, the predicted Δ*G* value is finally transformed into a value ranging from 0 to 1 using a sigmoid function. In the foldability prediction task, this transformed value was interpreted as the probability that the target protein folds into a unique and stable structure. *λ* is a scaling factor used to distinguish positive and negative Δ*G* values when foldability probabilities are calculated. In this study, *λ* was fixed at 10.0.
**Figure S7:** Predictive performance comparison between RINAMI models using the original or shuffled structural representations during inference. For inference with the data shuffle, we shuffled the concatenated structure‐based representations among variants with the same sequence length derived from the same wild‐type protein. This comparison aims to demonstrate that RINAMI uses variant‐specific structural information contained in ESMFold‐predicted structures, even among variants derived from the same wild‐type protein and having the same sequence length. The decrease in predictive performance after shuffling suggests that such variant‐specific structural representations contribute to Δ*G* prediction and that inaccuracies in predicted structures could propagate into Δ*G* prediction. For each evaluation metric, including Pearson's correlation coefficient (Pearson's *R*), Spearman's rank correlation coefficient (Spearman's *R*), root mean squared error (RMSE), and mean absolute error (MAE), the mean value is shown with error bars representing the standard deviation across the three cluster‐based data splits.
**Figure S8:** Predictive performance comparison between RINAMI models trained with and without multi‐task learning. The multi‐task model was trained using both the foldability prediction task and the Δ*G* regression task, whereas the ablated model was trained using only the Δ*G* regression task. (a) Comparison of Δ*G* prediction performance on the Mega‐scale test subdatasets. For each evaluation metric, the mean value is shown with error bars representing the standard deviation across the three cluster‐based data splits. (b) Comparison of Δ*G* prediction performance on the Maxwell dataset. Predictive performance was evaluated using Pearson's correlation coefficient (Pearson's *R*) and Spearman's rank correlation coefficient (Spearman's *R*), root mean squared error (RMSE), and mean absolute error (MAE).

## Data Availability

The Python code for RINAMI, training and testing scripts, model parameters, data splits, cluster definitions, and benchmark datasets are available at GitHub: https://github.com/NaokiTOMITA1221/RINAMI_PROTEIN_SCIENCE.git. A user‐friendly Google Colab implementation is also provided through the GitHub repository, allowing users to run RINAMI without local installation.
