# Supplementary material for: RINAMI: Residue‐attributed interpretable neural network for predicting absolute folding free energy by merging structure and sequence information
Source: Protein Sci. 2026 Jul 10;35(8):e70717. doi: 10.1002/pro.70717 (PMC13351927; doi:10.1002/pro.70717)
Supplement: Supplementary file 1 — Figure S1: Performance of Cagiada's method in predicting ΔG for natural and variant proteins. The left panel shows prediction results for a dataset consisting of proteins labeled as mut_type = “wt” from the Mega‐scale dataset and natural proteins from the Maxwell dataset. The right panel shows prediction results for variant proteins from the Mega‐scale dataset. The x‐ and y‐axes indicate the experimentally measured and predicted ΔG values, respectively. In each panel, Pearson's correlation coefficient (Pearson's R) and Spearman's rank correlation coefficient (Spearman's R) between predicted and experimentally measured ΔG values are shown in the bottom‐right text box. The low Pearson's R for variant proteins indicates that Cagiada's method shows poor predictive performance for variant ΔG prediction, whereas the Pearson's R for proteins in the left panel indicates moderate agreement with experimental trends. Table S1: Number of samples included in each subdataset. For each data split, the Mega‐scale dataset was partitioned using the clustering‐based method described in Section 4.1. “Extreme Positive/Negative Data” indicates entries labeled as “ΔG > 5 kcal/mol” or “ΔG < −1 kcal/mol,” which were used only for the foldability prediction task. “Numeric Data” indicates entries with numeric ΔG values, which were used for both the foldability prediction and ΔG regression tasks. Table S2: Predictive performance of RINAMI across three independent cluster‐based data splits and three independent training runs per split. Predictive performance is quantified using Pearson's correlation coefficient (Pearson's R) and Spearman's rank correlation coefficient (Spearman's R), root mean squared error (RMSE), and mean absolute error (MAE) between predicted and experimentally measured ΔG values. The first nine rows report the performance on the Mega‐scale test subdataset for each split–run combination. For each split, RINAMI was trained three times using different random initializations. M [file PRO-35-e70717-s001.docx]

**SUPPLEMENTARY INFORMATION**


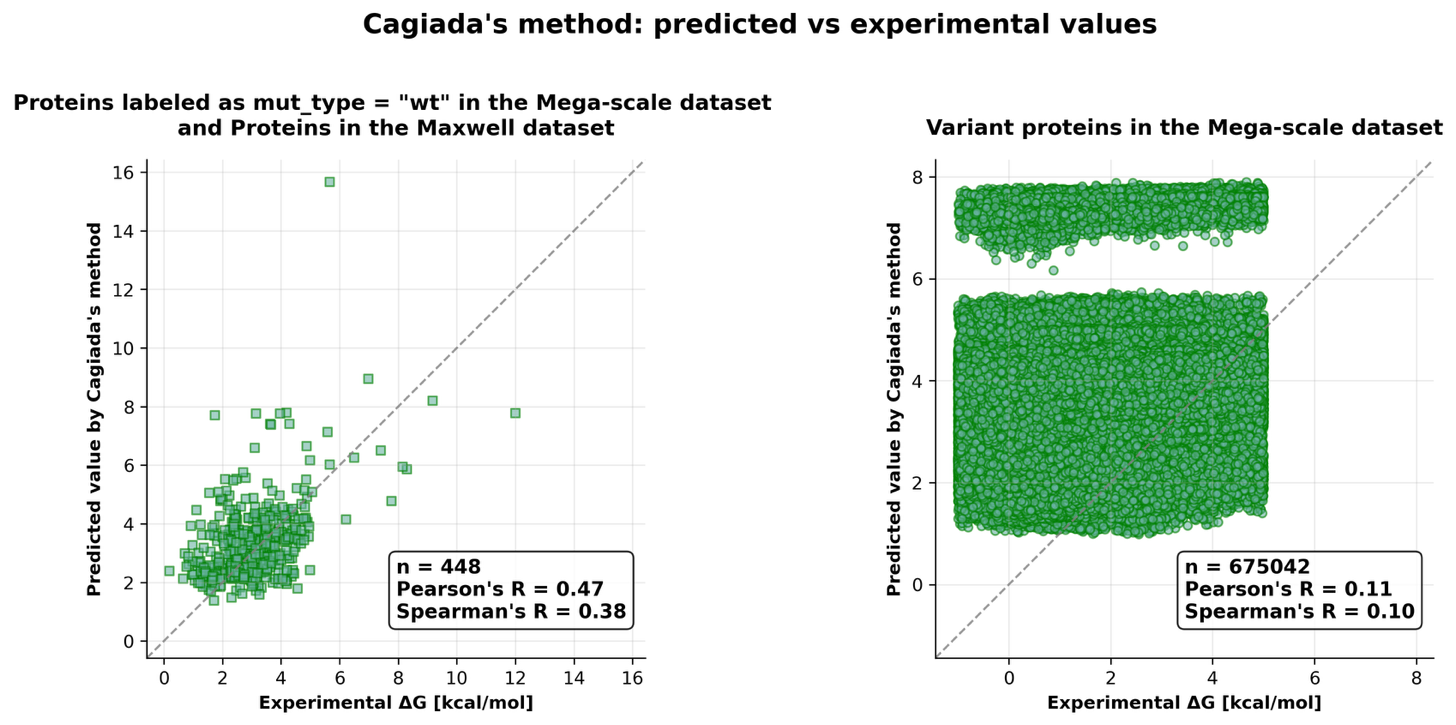


**FIGURE S1** Performance of Cagiada's method in predicting ΔG for natural and variant proteins. The left panel shows prediction results for a dataset consisting of proteins labeled as mut_type = "wt" from the Mega-scale dataset and natural proteins from the Maxwell dataset. The right panel shows prediction results for variant proteins from the Mega-scale dataset. The x- and y-axes indicate the experimentally measured and predicted ΔG values, respectively. In each panel, Pearson's correlation coefficient (Pearson's R) and Spearman's rank correlation coefficient (Spearman's R) between predicted and experimentally measured ΔG values are shown in the bottom-right text box. The low Pearson's R for variant proteins indicates that Cagiada's method shows poor predictive performance for variant ΔG prediction, whereas the Pearson's R for proteins in the left panel indicates moderate agreement with experimental trends.

**TABLE S1** Number of samples included in each subdataset. For each data split, the Mega-scale dataset was partitioned using the clustering-based method described in Section 4.1. "Extreme Positive/Negative Data" indicates entries labeled as "ΔG > 5 kcal/mol" or "ΔG < −1 kcal/mol," which were used only for the foldability prediction task. "Numeric Data" indicates entries with numeric ΔG values, which were used for both the foldability prediction and ΔG regression tasks.

**TABLE S2** Predictive performance of RINAMI across three independent cluster-based data splits and three independent training runs per split. Predictive performance is quantified using Pearson's correlation coefficient (Pearson's R) and Spearman's rank correlation coefficient (Spearman's R), root mean squared error (RMSE), and mean absolute error (MAE) between predicted and experimentally measured ΔG values. The first nine rows report the performance on the Mega-scale test subdataset for each split–run combination. For each split, RINAMI was trained three times using different random initializations. Mean values and standard deviations (Std) are shown for the models trained within each split, for all nine trained models, and for the lowest-performing models selected from the three splits. The "Average Normalized Metric Score" is a composite score used to identify the lowest-performing model; higher values indicate lower predictive performance. Details of its calculation are described in Section 2.2 and Section 4.4.

**TABLE S3** **Bootstrap analysis of predictive performance for each method on the test subdataset of each Mega-scale dataset split.** Predictive performance is quantified using Pearson's correlation coefficient (Pearson's R) and Spearman's rank correlation coefficient (Spearman's R), root mean squared error (RMSE), and mean absolute error (MAE) between predicted and experimentally measured ΔG values. For each analysis, 10,000 bootstrap iterations were performed. "Observed" indicates the metric value calculated using the full test subdataset before bootstrap resampling. "Bootstrap Mean" and "Bootstrap Std" indicate the mean and standard deviation of the metric values obtained from bootstrap resampling, respectively. "95%CI Low" and "95%CI High" indicate the lower and upper bounds of the 95% confidence interval, respectively. RMSE and MAE were not calculated for Rosetta energy scores because these scores are not expressed in [kcal/mol].

**TABLE S4** Bootstrap analysis of predictive performance on the external Maxwell dataset across all methods. Predictive performance is quantified using Pearson's correlation coefficient (Pearson's R) and Spearman's rank correlation coefficient (Spearman's R), root mean squared error (RMSE), and mean absolute error (MAE) between predicted and experimentally measured ΔG values. For each analysis, 10,000 bootstrap iterations were performed. "Observed" indicates the metric value calculated using the full Maxwell dataset before bootstrap resampling. "Bootstrap Mean" and "Bootstrap Std" indicate the mean and standard deviation, respectively, of the metric values obtained from bootstrap resampling. "95%CI Low" and "95%CI High" indicate the lower and upper bounds, respectively, of the 95% confidence interval. RMSE and MAE were not calculated for Rosetta energy scores because these scores are not expressed in [kcal/mol].

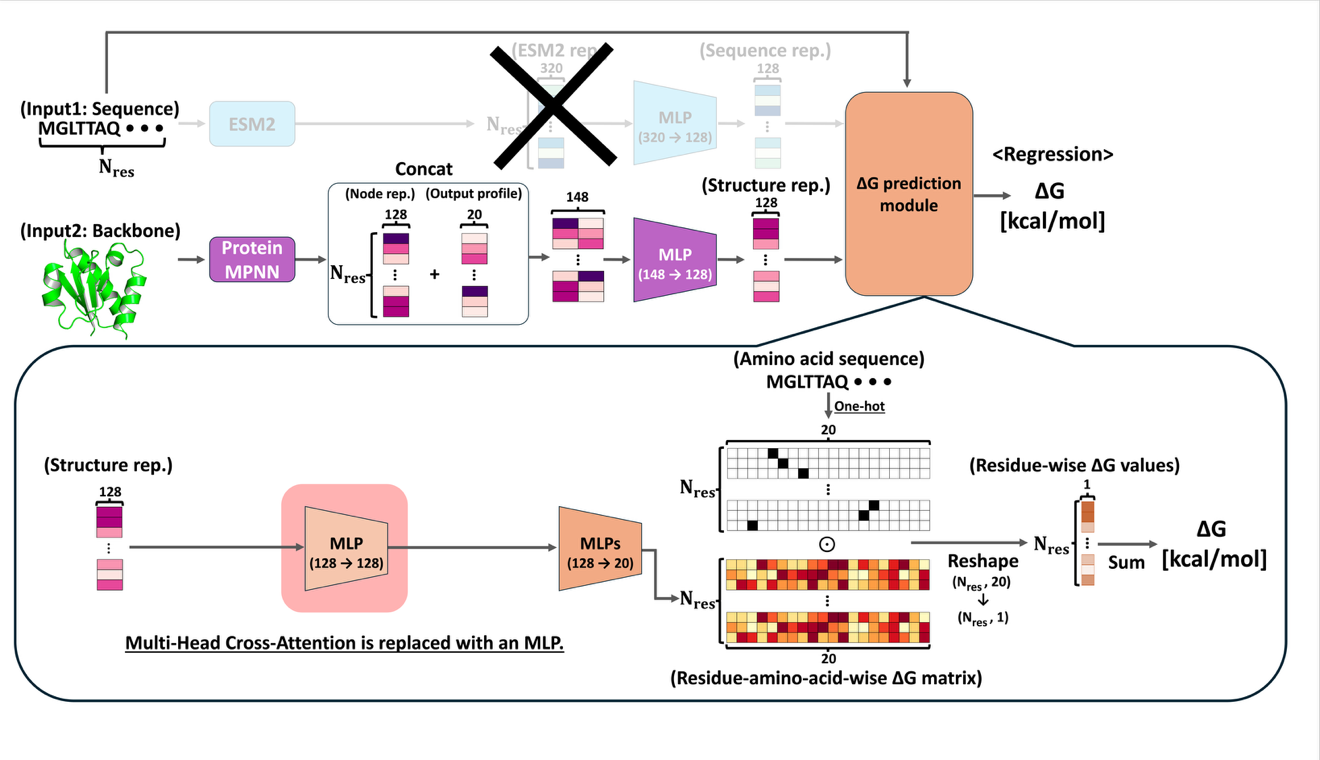


**FIGURE S2** Overview of the baseline model. In this model, the multi-head cross-attention module, which integrates the structural and sequence representations in RINAMI, is replaced with a multi-layer perceptron (MLP) that processes only the structural representation. This baseline model was used to evaluate the contribution of sequence–structure integration to ΔG prediction.


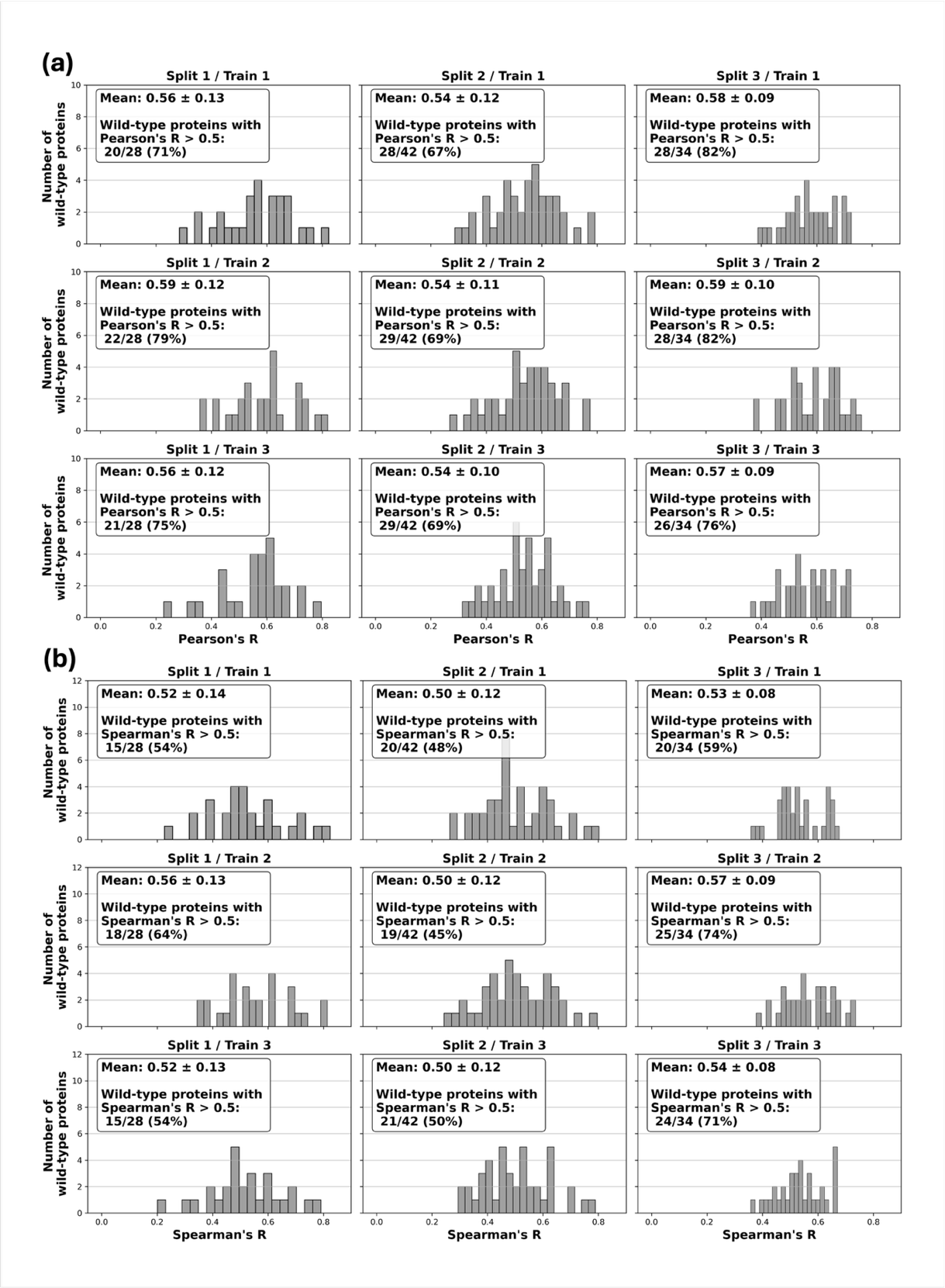


**FIGURE S3** Correlations between experimental single-mutation ΔΔG values and mutation-associated differences in partial ΔG scores across data splits and training runs. (a) Distributions of Pearson's correlation coefficient (Pearson's R) values calculated separately for each wild-type protein in the validation and test subdatasets of each split. These R values were calculated between experimentally measured ΔΔG values and the corresponding differences in partial ΔG scores for each single mutation. (b) Distributions of Spearman's rank correlation coefficient (Spearman's R) values calculated separately for each wild-type protein in the validation and test subdatasets of each split. These R values were calculated between experimentally measured ΔΔG values and the corresponding differences in partial ΔG scores for each single mutation.


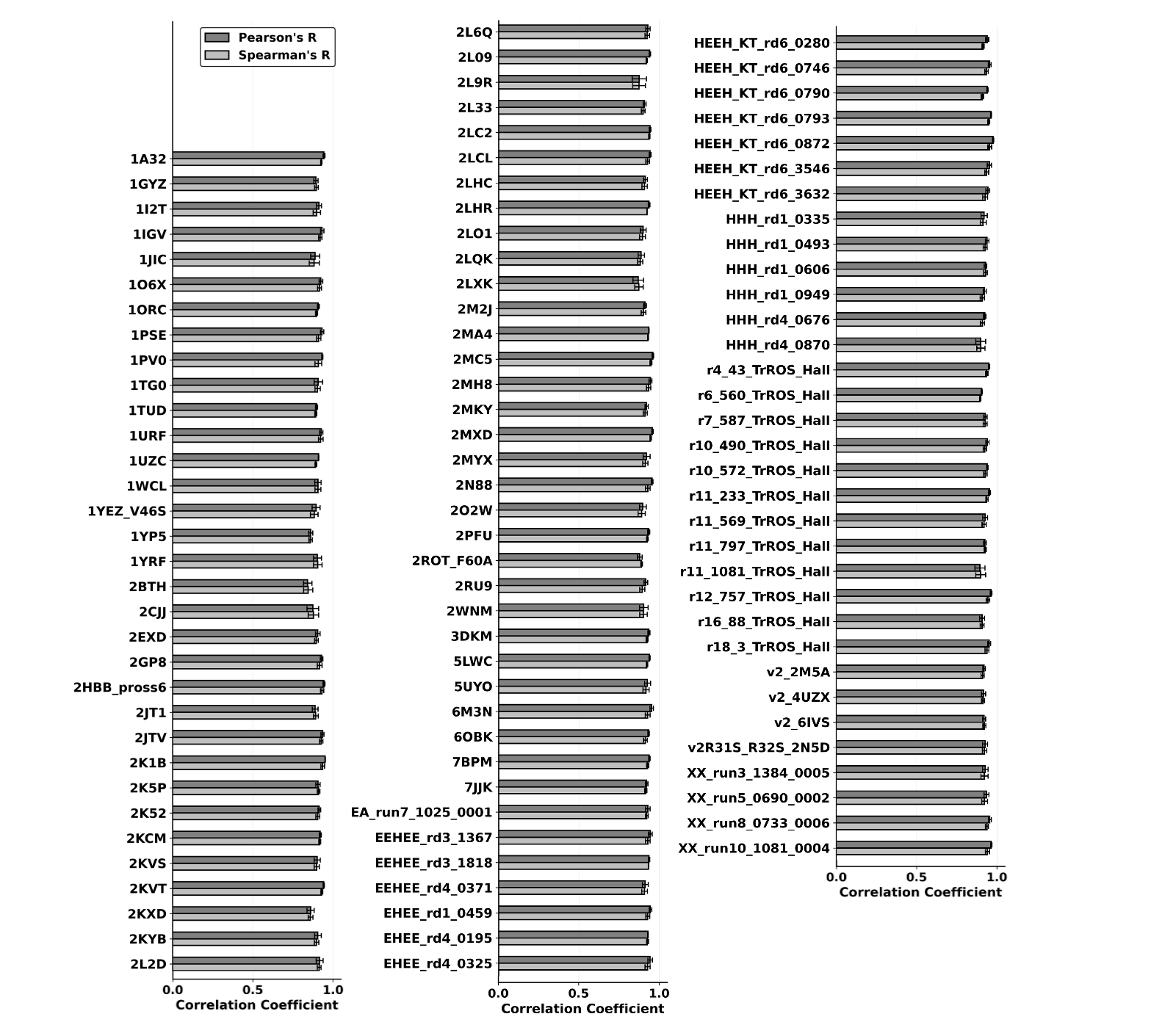


**FIGURE S4** Similarity of residue–amino-acid-wise ΔG matrices across independent training runs for each wild-type protein. For each wild-type protein included in the validation and test subdatasets of each split, residue–amino-acid-wise ΔG matrices derived from three independent training runs within the same split were compared in an all-versus-all manner. Horizontal bars represent the mean Pearson's correlation coefficient (Pearson's R) and Spearman's rank correlation coefficient (Spearman's R) values across the three pairwise comparisons, and error bars represent the standard deviations across these pairwise comparisons.


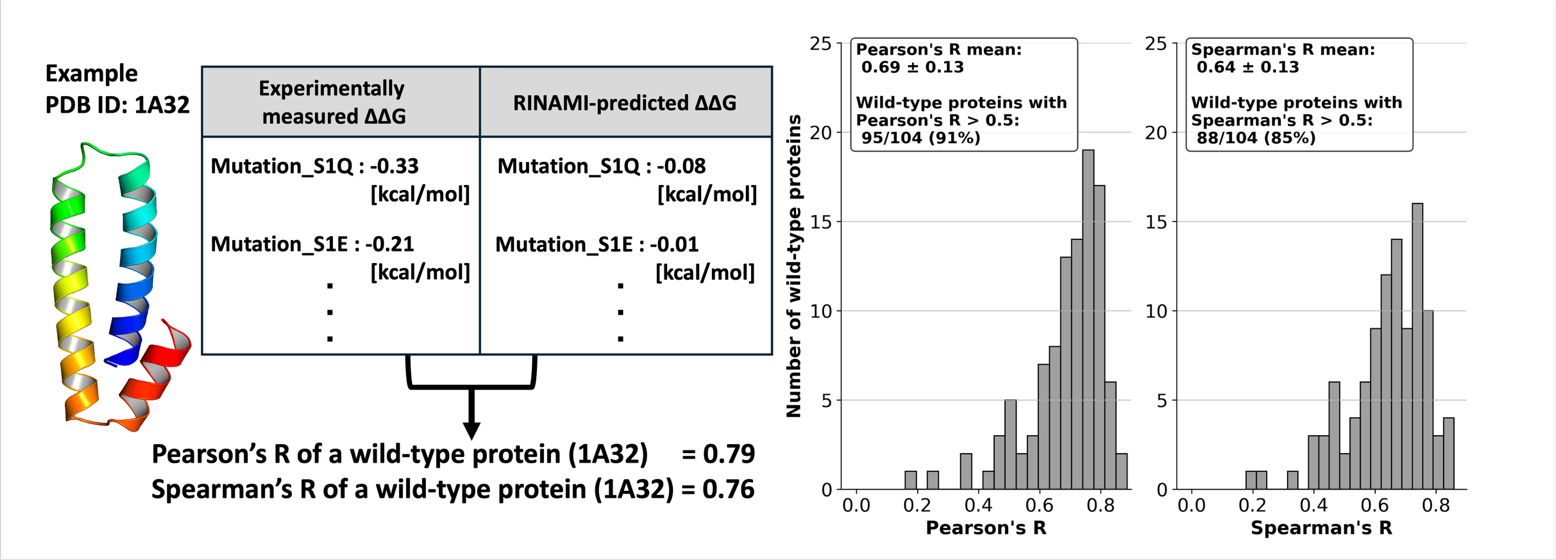


**FIGURE S5** Distributions of Pearson's correlation coefficient (Pearson's R) and Spearman's rank correlation coefficient (Spearman's R) values calculated separately for each wild-type protein between experimentally measured ΔΔG values and RINAMI-predicted ΔΔG values. **The left schematic illustrates the calculation procedure for one representative wild-type protein. For each wild-type protein, single-mutant variants derived from the same wild-type protein were collected, and a table was constructed containing the experimentally measured ΔΔG value and the RINAMI-predicted ΔΔG value for each mutation. The Pearson's R and Spearman's R values were then calculated across the single-mutant variants belonging to that wild-type protein.** Experimental ΔΔG values were calculated from the measured ΔG values of each single-mutant variant and its corresponding wild-type protein. RINAMI-predicted ΔΔG values were calculated from the predicted ΔG values of each single-mutant variant and its corresponding wild-type protein.


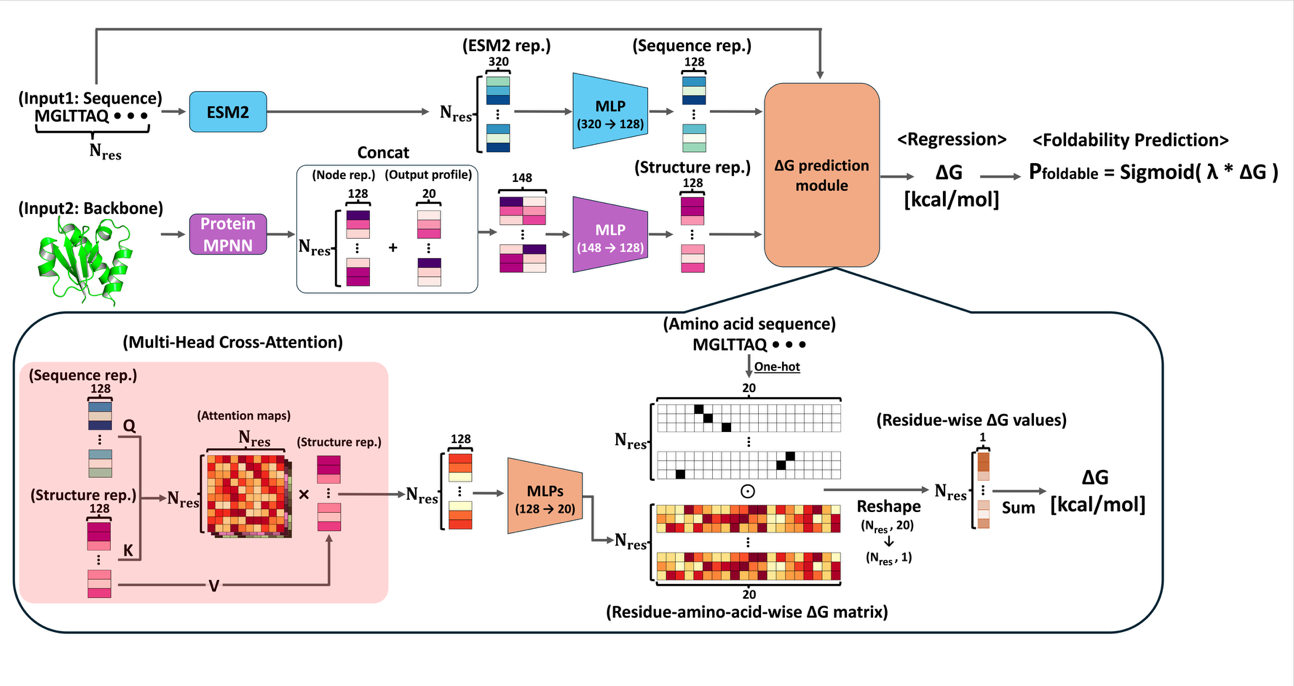


**FIGURE S6** Overview of the foldability prediction task. Most of the prediction process is the same as that depicted in Figure 1. In this architecture, the predicted ΔG value is finally transformed into a value ranging from 0 to 1 using a sigmoid function. In the foldability prediction task, this transformed value was interpreted as the probability that the target protein folds into a unique and stable structure. λ is a scaling factor used to distinguish positive and negative ΔG values when foldability probabilities are calculated. In this study, λ was fixed at 10.0.

**
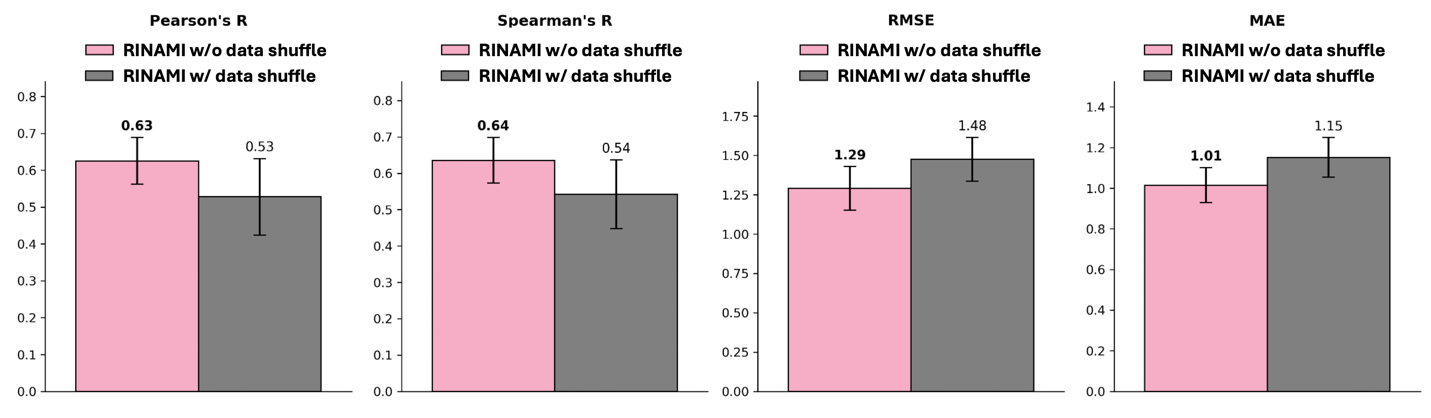
**

**FIGURE S7** Predictive performance comparison between RINAMI models using the original or shuffled structural representations during inference. For inference with the data shuffle, we shuffled the concatenated structure-based representations among variants with the same sequence length derived from the same wild-type protein. This comparison aims to demonstrate that RINAMI uses variant-specific structural information contained in ESMFold-predicted structures, even among variants derived from the same wild-type protein and having the same sequence length. The decrease in predictive performance after shuffling suggests that such variant-specific structural representations contribute to ΔG prediction and that inaccuracies in predicted structures could propagate into ΔG prediction. For each evaluation metric, including Pearson's correlation coefficient (Pearson's R), Spearman's rank correlation coefficient (Spearman's R), root mean squared error (RMSE), and mean absolute error (MAE), the mean value is shown with error bars representing the standard deviation across the three cluster-based data splits.

**
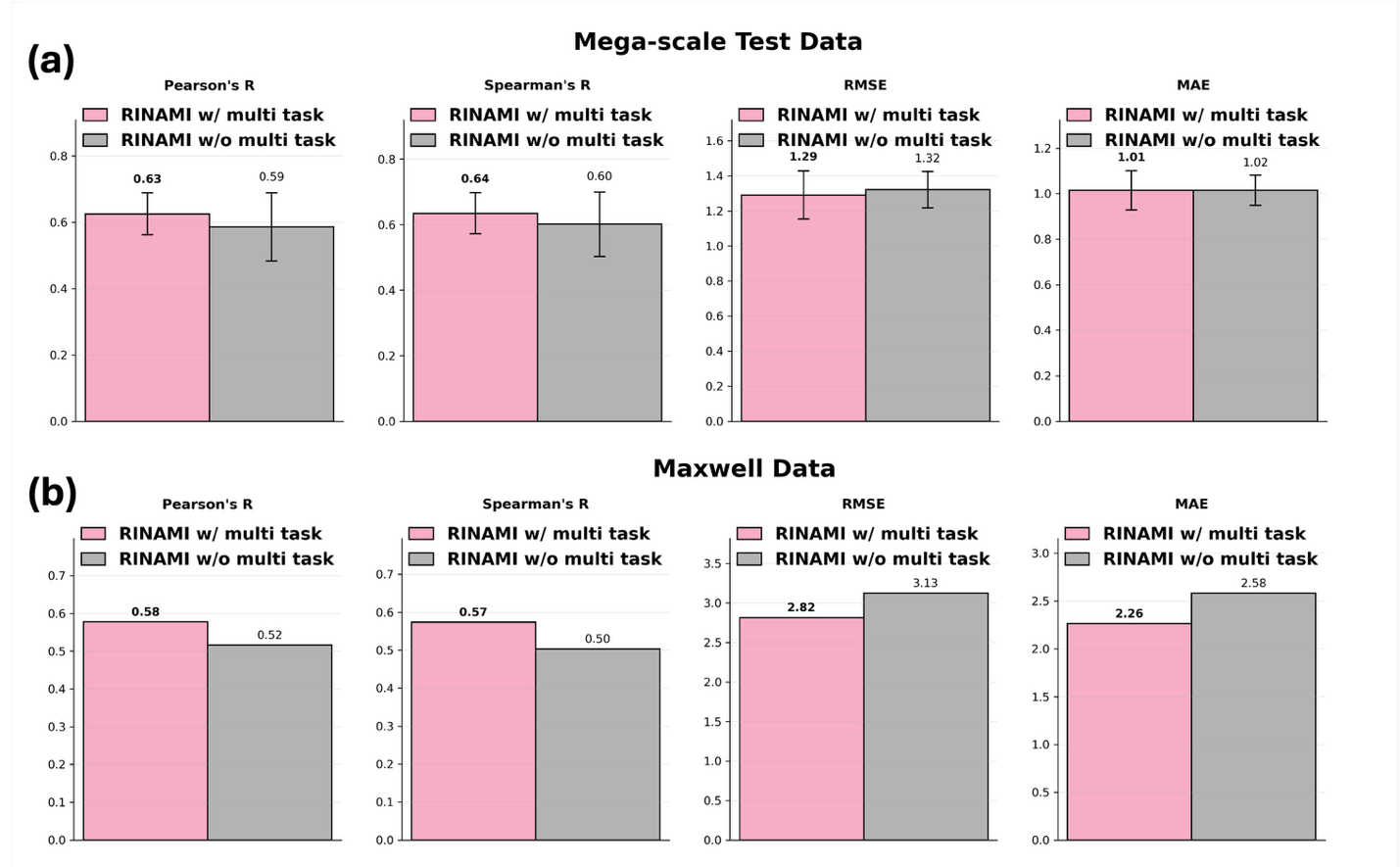
**

**FIGURE S8** Predictive performance comparison between RINAMI models trained with and without multi-task learning. The multi-task model was trained using both the foldability prediction task and the ΔG regression task, whereas the ablated model was trained using only the ΔG regression task. (a) Comparison of ΔG prediction performance on the Mega-scale test subdatasets. For each evaluation metric, the mean value is shown with error bars representing the standard deviation across the three cluster-based data splits. (b) Comparison of ΔG prediction performance on the Maxwell dataset. Predictive performance was evaluated using Pearson's correlation coefficient (Pearson's R) and Spearman's rank correlation coefficient (Spearman's R), root mean squared error (RMSE), and mean absolute error (MAE).
